# Intracellular Ca^2+^ Modulates PKA Compartmentalization and Dynamics in Human iPSC-Derived Cardiomyocytes

**DOI:** 10.3390/ijms27156559

**Published:** 2026-07-23

**Authors:** Anat Rotschield, Savyon Mazgaoker, Sofia Segal, Ido Weiser-Bitoun, Inbar Brosh, Ofer Binah, Yael Yaniv

**Affiliations:** 1 Laboratory of Bioelectric and Bioenergetic Systems, Faculty of Biomedical Engineering, Technion-IIT, Haifa 3200003, Israel; anat.ro@campus.technion.ac.il (A.R.); broshi@gmail.com (I.B.); 2 Department of Internal Medicine “C”, Rambam Health Care Campus, Haifa 3109601, Israel; 3 The Leir Clinical Research Institute at Rambam (CRIR), Rambam Health Care Campus, Haifa 3109601, Israel; 4 Department of Physiology, Biophysics and Systems Biology, Ruth and Bruce Rappaport Faculty of Medicine and Research Institute, Technion-IIT, Haifa 3525433, Israel; binah@technion.ac.il

**Keywords:** cyclic adenosine monophosphate (cAMP), protein kinase A (PKA), pacemaker, PKA compartmentalization, hiPSC-CMs, sinoatrial node cells (SANCs)

## Abstract

The automaticity of human-induced Pluripotent Stem Cell-derived cardiomyocytes (hiPSC-CMs) is governed by coupled Ca^2+^ and membrane clocks, coordinated through local Ca^2+^ releases (LCRs) and cyclic adenosine monophosphate (cAMP)/protein kinase A (PKA) signaling. We investigated the role of PKA in hiPSC-CM energetics by measuring its dynamics in the cytosol and mitochondria, and its crosstalk with Ca^2+^. We tested three hypotheses: (i) Ca^2+^-activated PKA signaling regulates energy balance; (ii) adenylyl cyclase activity correlates with spontaneous beating; and (iii) PKA compartmentalization is Ca^2+^-dependent. We also compared hiPSC-CMs with rabbit sinoatrial node cells (SANCs). The key findings are: (i) PKA inhibition (H-89), Ca^2+^ chelation (BAPTA), or mitochondrial Ca^2+^ blockade (Ru360) led to energy imbalance; (ii) H-89 induced compartmentalized PKA activity in the cytosol, mitochondrial matrix, and outer mitochondrial membrane; (iii) Ca^2+^ chelation with BAPTA reduced PKA activity globally; and (iv) PKA dynamics and Ca^2+^-dependent regulation were similar in hiPSC-CMs and rabbit SANCs. In conclusion, intracellular Ca^2+^-mediated PKA compartmentalization is present in hiPSC-CMs and rabbit SANCs.

## 1. Introduction

The rate and rhythm of spontaneously beating human induced Pluripotent Stem Cell-derived cardiomyocytes (hiPSC-CMs) are regulated by the Ca^2+^ and membrane (M) clocks [1]. In this regard, we recently demonstrated in hiPSC-CMs that the coupling between these clocks is regulated by sarcoplasmic reticulum (SR)-originated local Ca^2+^ releases (LCRs) and cyclic adenosine monophosphate (cAMP)/protein kinase A (PKA) signaling [2]. Further, the LCRs were shown to couple the two clocks by activating the M clock to generate action potentials [3,4,5,6]. Importantly, while LCRs [2] and cytosolic cAMP [7] dynamics were reported in hiPSC-CMs, PKA spatiotemporal dynamics and correlation to hiPSC-CMs spontaneous activity were not investigated. Not only is Ca^2+^ in the SR important, but mitochondrial Ca^2+^ was also shown to play a crucial role in regulating the rate and rhythm of hiPSC-CMs [8]. Moreover, the crosstalk between PKA and Ca^2+^ is also important for matching ATP supply to demand in hiPSC-CMs [9,10].

Because spontaneously beating hiPSC-CMs have shown similarities to sinoatrial node cells (SANCs) respecting action potential morphology, control mechanisms, and energetics, a comparison with mammalian SANCs, specifically from rabbits, is of importance [2,10]. As previously reported, mammalian SANC function is also controlled by both Ca^2+^ and M clocks [11]. In SANCs, Ca^2+^ and cAMP/PKA mutually entrain each other, leading to high PKA activity [12] due to the presence of Ca^2+^-activated adenylyl cyclase (AC; AC1 and AC8) [13,14]. The reports that AC1 is present in microdomains of mouse SANCs [15] and that Ca^2+^ activates AC suggest that PKA dynamics vary across distinct compartments. While AC1 has also been reported in hiPSC-CMs [16], it is still unknown whether PKA compartmentation exists. In addition, PKA signaling was shown to be an important regulator of ATP supply to demand matching in SANCs [17]. However, since it is still unknown whether PKA has a significant role in the energetic balance of hiPSC-CMs, measurements of PKA in the mitochondria should be conducted. Because Ca^2+^ dynamics differ in the cytosol versus mitochondria [18], it is plausible that if indeed Ca^2+^ activates AC, PKA dynamics should differ between the cytosol and the mitochondrial microdomains. Further, although in SANCs cAMP has dissimilar dynamics in distinct compartments [15], PKA spatiotemporal dynamics were not measured. While cytosolic PKA activity was measured in rabbit SANCs [19], these cultured SANCs underwent rapid remodeling respecting their morphology and electrophysiological properties [20]. Therefore, to properly compare PKA spatial dynamics in hiPSC-CMs and SANCs in cytosolic and mitochondrial compartments, reliable measurements should also be performed in the latter.

Understanding how signaling pathways coordinate energy balance in human cardiomyocytes remains a major challenge in cardiac biology, particularly in the context of human iPSC-CMs, which are widely used for disease modeling, drug testing, and regenerative medicine. In these cells, the interplay between Ca^2+^ signaling and cAMP/PKA pathways is thought to be critical for regulating contractile activity and metabolic demand, yet their spatiotemporal organization and functional coupling remain poorly defined. To investigate the spatiotemporal dynamics of PKA in hiPSC-CMs, its regulation of energy balance, and its modulation by Ca^2+^, we investigated three hypotheses: (i) Ca^2+^-activated PKA-dependent signaling is an important regulator of energy balance; (ii) the activity of the Ca^2+^-activated ACs correlates with the spontaneously beating rate of hiPSC-CMs; and (iii) PKA compartmentalization is regulated by intracellular Ca^2+^ concentration. To measure the basal PKA dynamics and their pharmacological modulation in hiPSC-CMs, we employed fluorescence resonance energy transfer (FRET)-based PKA activity sensors targeted to the cytosol, outer mitochondrial membrane (OMM), and mitochondrial matrix (MM). Our study shows that PKA activity is differentially compartmentalized in the MM and OMM compared with the cytosol, and that Ca^2+^ plays a key role in the compartmentalization of PKA activity.

## 2. Results

### 2.1. The Role of Ca^2+^-Activated, PKA-Dependent Signaling in hiPSC-CMs

**Figure 1:**
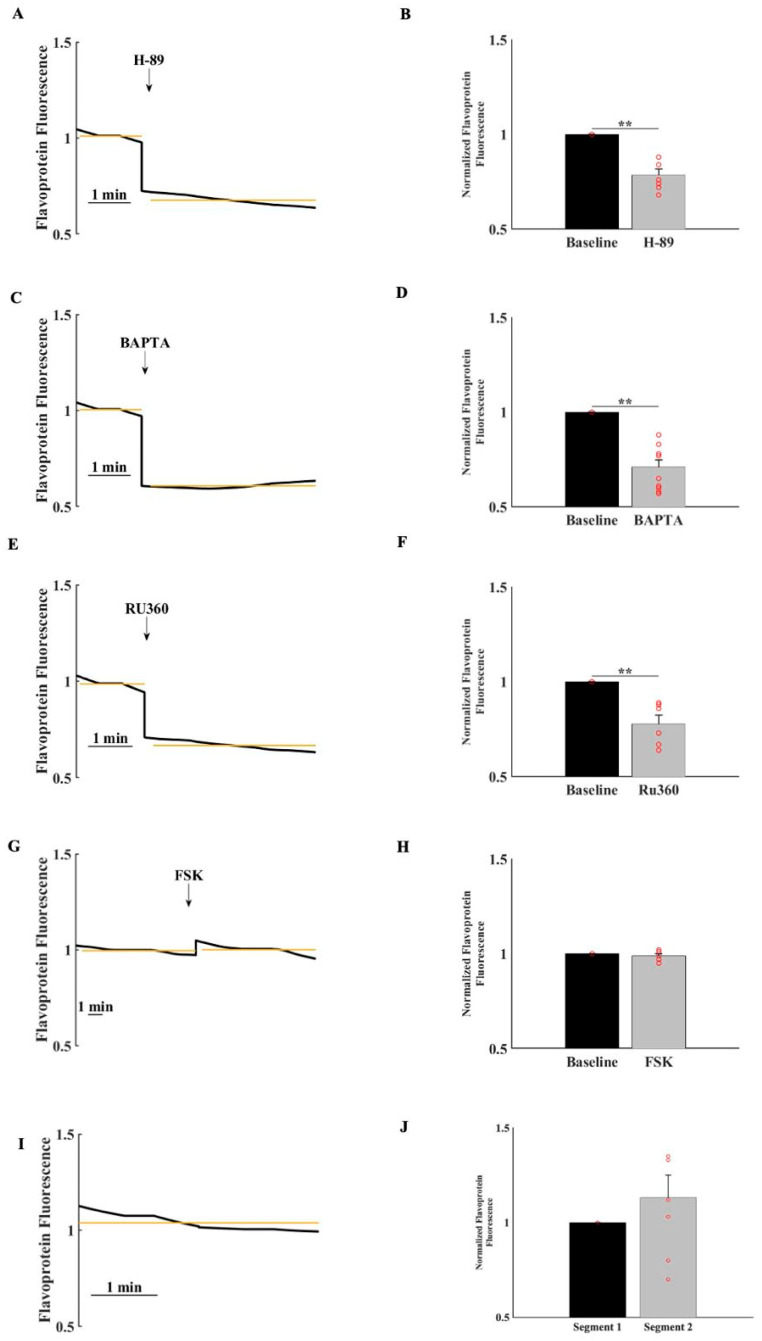
Flavoprotein fluorescence of spontaneously beating hiPSC-CMs. Representative (black traces) and mean (yellow line) flavoprotein fluorescence, in response to: (**A**,**B**) 10 µM H-89 (*n* = 7), (**C**,**D**) 25 µM BAPTA (*n* = 10), (**E**,**F**) 20 µM Ru360 (*n* = 6), (**G**,**H**) 20 µM forskolin (*n* = 6), and (**I**,**J**) time-control response (*n* = 7). Data are presented as mean ± SEM. ** *p* < 0.0001, H-89, BAPTA, or Ru360 compared with a value of 1.0 using a one-sample *t*-test.

To investigate the role of Ca^2+^-activated PKA cascades in energy balance, the effect of this cascade inhibition on the cell and mitochondria was assessed. Flavoprotein fluorescence was reduced following application of 10 µM of the PKA inhibitor H-89 (Figure 1A), 25 µM of the Ca^2+^ chelator BAPTA (Figure 1B), or 20 µM of the mitochondrial Ca^2+^ inhibitor Ru360 (Figure 1C). In contrast, 20 µM of the PKA activator forskolin (FSK, Figure 1D) or DMSO treatment (Figure 1E) did not affect flavoprotein fluorescence.

### 2.2. Cytosolic PKA Dynamics in hiPSC-CMs

**Figure 2:**
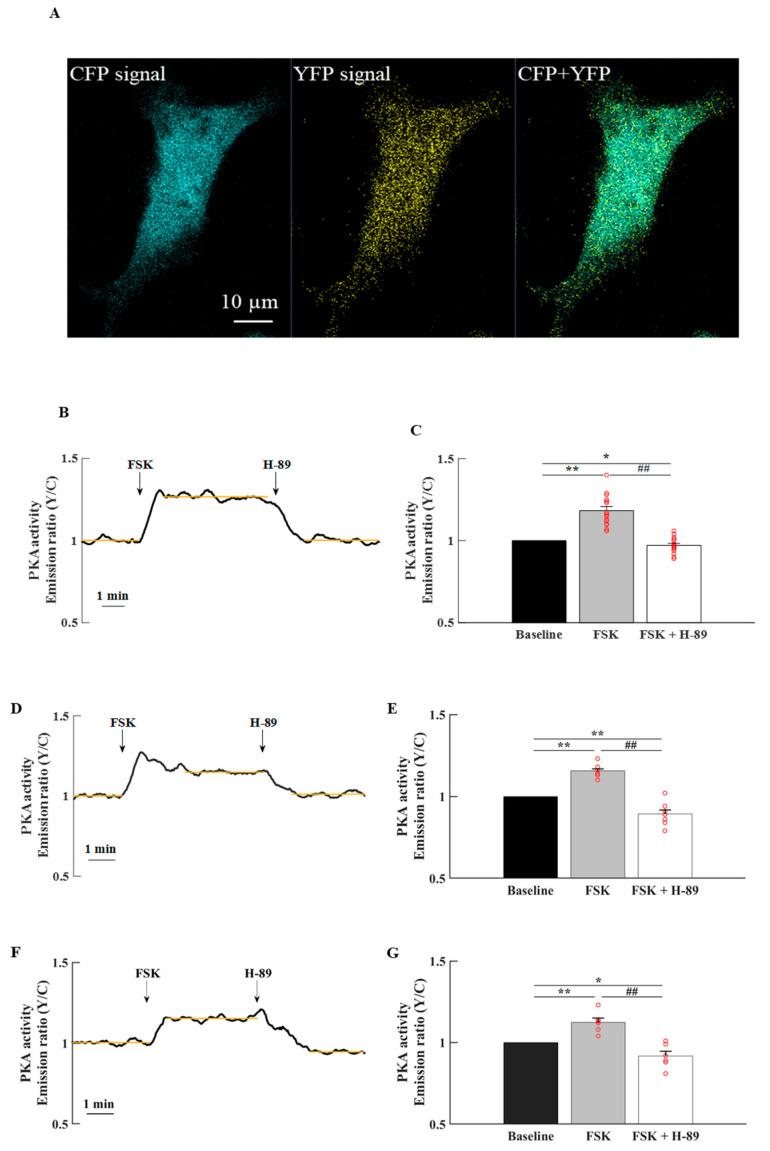
PKA activity in the cytosol and in the mitochondrial compartments of spontaneously beating hiPSC-CMs. (**A**) A confocal image of the CFP and YFP signal in a single hiPSC-CM expressing pcDNA3-AKAR4. (**B**) A representative example of cytosolic PKA activity (AKAR4 emission Y/C) in a single hiPSC-CM in response to adenylyl cyclase (AC) activation with 20 µM forskolin (FSK), followed by PKA inhibition using 10 µM H-89. (**C**) The mean PKA activity in the cytosol in response to FSK and H-89 (*n* = 15). (**D**) A representative example of PKA activity in the outer mitochondrial membrane (OMM) (yTom -AKAR4 emission Y/C) in a single hiPSC-CM in response to adenylyl cyclase (AC) activation using 20 µM forskolin (FSK), followed by PKA inhibition with 10 µM H-89. (**E**) The mean PKA activity in the OMM in response to FSK and H-89 (*n* = 8). (**F**) A representative example of mitochondrial matrix (MM) PKA activity (4Cox8-AKAR4 emission Y/C) in a single hiPSC-CM in response to 20 µM FSK, followed by 10 µM H-89. (**G**) The mean PKA activity in the MM in response to FSK and H-89 (*n* = 6). The data are presented as the mean ± SEM. * *p* < 0.05, ** *p* < 0.01, andcompared with a value of 1.0 using a one-sample *t*-test. ## *p* < 0.01 FSK versus H-89 using a paired Student’s *t*-test.

PKA activity was measured using fluorescence resonance energy transfer (FRET) AKAR probes (see Materials and Methods Section 4, Figure 2A). To determine the absolute PKA activity in the cytosol, the activity was compared to the maximal and minimal activities measured with FRET probes (independent of the flavoprotein fluorescence experiments). Baseline PKA activity was increased by 20 µM of the AC activator FSK (Figure S1A,B), but was not further augmented by 40 µM FSK. Administering the phosphodiesterase (PDE) inhibitor 3-isobutyl-1-methylxanthine (IBMX, 100 µM) after 20 µM FSK did not further increase PKA activity (Figure S1C,D), indicating that maximum PKA activity was already attained by FSK. PKA activity was decreased by 10 µM of the PKA inhibitor H-89 (Figure S2A,B). Administering MDL (400 µM), which inhibits AC activity after 10 µM H-89, did not further decrease PKA activity (Figure S2C,D), indicating that minimum PKA activity was already attained with H-89. PKA activity in the cytosol was increased by 20 µM FSK and decreased by 10 µM H-89 (Figure 2B). Specifically, basal PKA activity was increased by FSK from 1.0 to 1.18 ± 0.02, and decreased by H-89 to 0.97 ± 0.01 (Figure 2C). When normalized to the activities measured in response to FSK (assumed to be 100%) and H-89 alone (assumed to be 0), basal PKA activity in the cytosol was 0.13 ± 0.06.

Because AKAR readouts reflect the net balance between PKA-mediated phosphorylation and phosphatase-mediated dephosphorylation, changes in FRET signal cannot be interpreted as a direct, isolated measure of PKA activity without considering phosphatase contributions. Baseline PKA activity was not altered by 100 nM of the PP2A inhibitor okadaic acid (OA; Figure S3A,B). Although no change in PKA activity was detected, Figure S4 shows that the beating rate increased following treatment with 100 nM OA. In response to the PP1 inhibitor calyculin A, no increase in the beating rate was observed across concentrations range of 1-500 nM. Thus, phosphatases have a minor effect on PKA activity in hiPSC-CMs. To further assess whether PDE inhibition, specifically PDE1 inhibition, affects PKA activity, we used the PDE1 inhibitor MIMX, followed by treatment with IBMX. MIMX did not alter PKA activity, whereas IBMX increased it (Figure S5).

### 2.3. PKA Compartmentalization in the Cytosol, MM, and OMM in hiPSC-CMs

To determine whether PKA penetrates the MM in hiPSC-CMs, as was documented in HeLa and HEK293 cells [21] and atrial cells [22], hiPSC-CMs were loaded with OMM- and MM-targeted sensors. Similar to the cytosol, baseline PKA activity was increased by 10 µM FSK (Figures S6 and S7 for the OMM and MM, respectively), but was not further augmented by 20 and 40 µM FSK. PKA activity decreased by 10 µM of the PKA inhibitor H-89 (Figures S6 and S7 for the OMM and MM, respectively). Administering MDL-12,330A (MDL, 400 µM) after 10 µM H-89 did not further decrease PKA activity (Figures S6 and S7 for the OMM and MM, respectively), indicating that minimum PKA activity was already attained with H-89.

Basal PKA activity in the OMM increased from 1.0 to 1.15 ± 0.01 by 20 µM FSK and decreased by 10 µM H-89 to 0.89 ± 0.02 (Figure 2D,E), demonstrating that PKA activity is also present in the mitochondria. Similar responses to FSK and H-89 were measured in the MM (Figure 2F), where PKA activity increased from 1.0 to 1.12 ± 0.02 and decreased to 0.91 ± 0.03, respectively (Figure 2G). The FSK-mediated increase in PKA activity was similar (*p* = 0.2844) in all compartments, while the responses to H-89 in the cytosol and MM were similar (*p* = 0.1986), but lower (*p* < 0.05) than in the OMM, suggesting that PKA response to H-89 differs across intracellular compartments. Normalized basal PKA activity values (to minimal and maximal values in the specific compartment) were higher (*p* < 0.05) in the OMM (0.37 ± 0.08) and MM (0.32 ± 0.13) compared with the cytosol.

### 2.4. Ca^2+^-Activated AC Is an Important Regulator of PKA Activity in hiPSC-CMs

**Figure 3:**
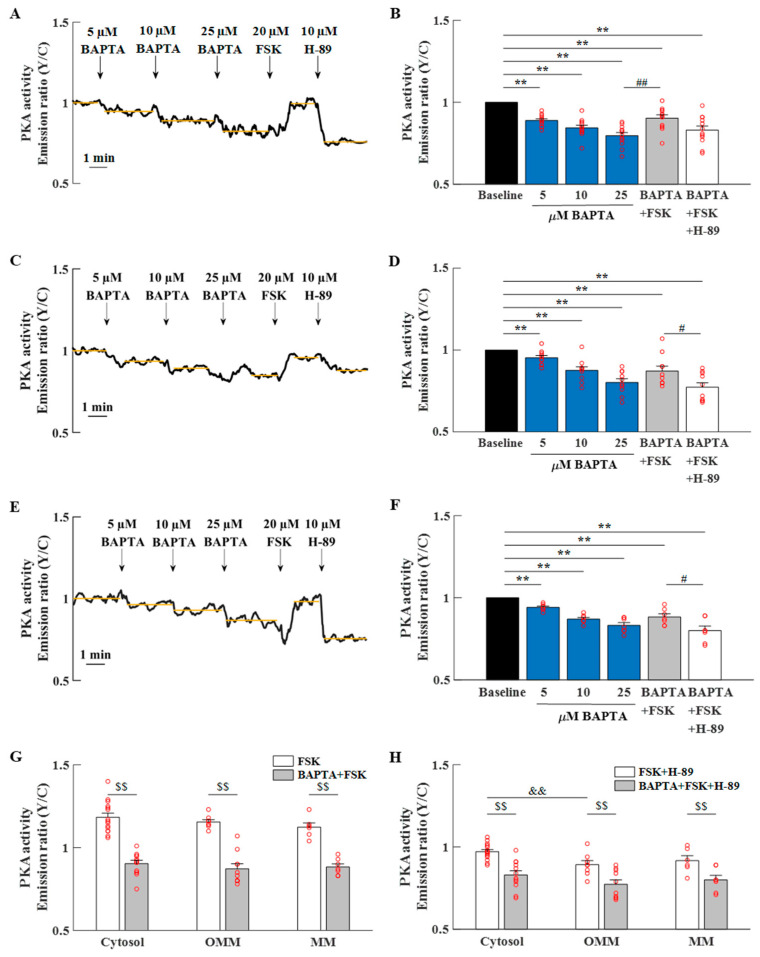
PKA activity in the cytosol and in mitochondrial compartments of spontaneously beating hiPSC-CMs. (**A**) A representative example of cytosolic PKA activity (AKAR4 emission Y/C) in a single hiPSC-CM in response to increasing concentrations of a Ca^2+^ chelator, BAPTA (5, 10, and 25 µM), followed by adenylyl cyclase (AC) activation using 20 µM forskolin (FSK) and PKA inhibition using 10 µM H-89. (**B**) The mean PKA activity in the cytosol in response to increasing concentrations of BAPTA, FSK, and H-89 (*n* = 12). (**C**) A representative example of PKA activity in the outer mitochondrial membrane (OMM, yTom-AKAR4 emission Y/C) in a single hiPSC-CM in response to increasing concentrations of BAPTA (5, 10, and 25 µM), followed by 20 µM FSK and 10 µM H-89. (**D**) The mean PKA activity in the OMM in response to increasing concentrations of BAPTA, FSK, and H-89 (*n* = 10). (**E**) A representative example of mitochondrial matrix (MM) PKA activity (4Cox8-AKAR4 emission Y/C) in a single hiPSC-CM in response to increasing concentrations of BAPTA (5, 10, and 25 µM), followed by 20 µM FSK and 10 µM H-89. (**F**) The mean PKA activity in the MM in response to increasing concentrations of BAPTA, FSK, and H-89 (*n* = 7). (**G**) PKA activity in the cytosol, OMM, and MM of a single hiPSC-CM following coadministration of BAPTA (25 µM) and FSK (20 µM). (**H**) PKA activity in the cytosol, OMM, and MM of a single hiPSC-CM following coadministration of BAPTA (25 µM) and H-89 (10 µM). The data are presented as the mean± SEM. ** *p* < 0.01, BAPTA, FSK, or H-89 compared with a value of 1.0, using a one-sample *t*-test. # *p* < 0.05 and ## *p* < 0.01compared among BAPTA, FSK, and H-89, using a one-way ANOVA with Dunn–Šidák multiple comparison. $$ *p* < 0.01 FSK or H-89 versus the response in the presence of BAPTA in the same compartment, and && *p* < 0.01 FSK or H-89 versus the response in the presence of BAPTA in the different compartment, using an unpaired Student’s *t*-test, and *p* < 0.05 compared among H-89 responses in the cytosol, OMM, and MM using a one-way ANOVA with Dunn–Šidák multiple comparison.

To determine whether Ca^2+^ regulates PKA activity, we first confirmed the presence of Ca^2+^-activated AC1 (Figure S8A) and AC8 (Figure S8B) in hiPSC-CMs, using anti-AC1 and anti-AC8 antibodies (*n* = 10 for each AC type). Next, hiPSC-CMs were treated with BAPTA to chelate cytosolic Ca^2+^. Figure S9 shows a representative example. As expected on average, application of BAPTA decreased the beating rate, *p* = 0.0001 < 0.05, as well as the Ca^2+^ transient kinetics (T_50_ comparison between baseline and BAPTA treatment with a paired *t*-test was *p* = 0.0205 < 0.05; T_90_ was *p* = 0.0231 < 0.05 and time to peak was *p* = 0.0245 < 0.05). Figure 3A shows a representative example of the effects of increasing the BAPTA concentration on PKA activity in the cytosol. Compared to control, 25 µM BAPTA reduced PKA activity by 21 ± 1.8% (Figure 3B). Similar effects were measured in the OMM (Figure 3C,D) and MM (Figure 3E,F), with reductions of 20 ± 2.2% and 17 ± 1.7%, respectively. Note that the level of PKA activity after 25 µM BAPTA was not different from that observed with H-89 alone or with H-89 combined with BAPTA in all compartments (Figure 3). The observed reduction in PKA activity by BAPTA suggests that Ca^2+^ modulates PKA activity in different intracellular compartments.

To determine whether reduced Ca^2+^ concentration limits the maximal PKA activity caused by AC, the activity was stimulated by adding FSK following BAPTA treatment. This dual treatment resulted in lower PKA activity (*p* < 0.00001) in the cytosol (0.9 ± 0.02 compared with 1.18 ± 0.02 in FSK alone), in the OMM (0.87 ± 0.03 compared with 1.15 ± 0.01 in FSK alone) and in the MM (0.88 ± 0.01 vs. 1.12 ± 0.02 in FSK alone), compared with the maximal PKA activity obtained by adding only FSK (Figure 3G). These findings suggest that Ca^2+^ indeed regulates PKA activity in these compartments. To ensure that the effect of BAPTA on PKA activity was assessed at a steady state, we applied 25 µM BAPTA and waited 5 min. The reduction in PKA activity induced by BAPTA (Figure S10) across all compartments was similar to that observed in the BAPTA dose–response analysis. Coadministration of BAPTA and FSK resulted in similar (*p* = 0.619) PKA activity in the MM and OMM compared with the cytosol. The dual treatment with BAPTA and H-89 resulted in lower PKA activity in the cytosol (0.83 ± 0.02 vs. 0.97 ± 0.01 in the H-89 control), OMM (0.77 ± 0.02 vs. 0.89 ± 0.02 in the H-89 control), and in the MM (0.8 ± 0.02 vs. 0.91 ± 0.03 in the H-89 control), compared with the minimal PKA activity generated by PKA inhibition with H-89 (Figure 3H). Coadministration of BAPTA and H-89 resulted in a similar (*p* = 0.2941) PKA activity in the MM and OMM compared to the cytosol. The Ca^2+^-dependent effect of H-89 further supports the notion that Ca^2+^ affects PKA compartmentation.

### 2.5. Mitochondrial Ca^2+^-Activated AC Is an Important Regulator of PKA Activity in hiPSC-CMs

**Figure 4:**
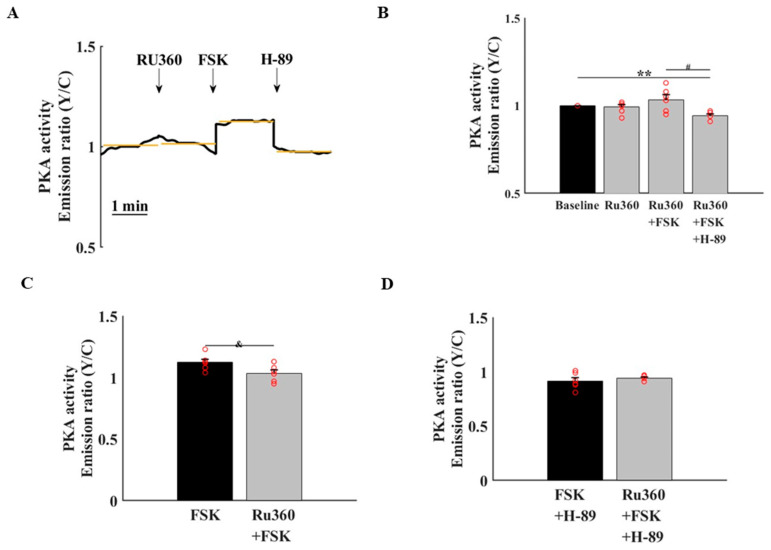
The effect of the mitochondrial Ca^2+^ blocker Ru360 on PKA activity in the mitochondrial matrix of spontaneously beating hiPSC-CMs. (**A**) A representative example of mitochondrial matrix PKA activity in a single spontaneously beating hiPSC-CM in response to the mitochondrial Ca^2+^ blocker Ru360 (20 µM), followed by adenylyl cyclase (AC) activation with 20 µM forskolin (FSK) and PKA inhibition with 10 µM H-89. (**B**) The mean PKA activity in the mitochondrial matrix (4Cox8-AKAR4 emission Y/C) in a single spontaneously beating hiPSC-CM in response to the mitochondrial Ca^2+^ blocker Ru360 (20 µM), followed by adenylyl cyclase (AC) activation with 20 µM forskolin (FSK) and PKA inhibition with 10 µM H-89 (*n* = 6). (**C**) The mean PKA activity in the mitochondrial matrix (4Cox8-AKAR4 emission Y/C) of a single spontaneously beating hiPSC-CM following coadministration of Ru360 (20 µM) and FSK (20 µM (*n* = 6) or FSK alone (*n* = 6). (**D**) The mean PKA activity in the mitochondrial matrix (4Cox8-AKAR4 emission Y/C) of a single spontaneously beating hiPSC-CM following coadministration of Ru360 (20 µM) and H-89 (10 µM) (*n* = 6) or H-89 alone (*n* = 6). The data are presented as the mean ± SEM of ** *p* < 0.01, # *p* < 0.05 Ru360, FSK, or H-89 compared with a value of 1.0 using a one-sample *t*-test and compared among Ru360, FSK, and H-89 using a one-way ANOVA with Dunn–Šidák multiple comparison. & *p* < 0.05 FSK or H-89 versus the response in the presence of Ru360 using an unpaired *t*-test.

To determine whether mitochondrial Ca^2+^ regulates PKA activity, hiPSC-CMs were treated with Ru360 to inhibit Ca^2+^ influx into the mitochondria. Figure 4A shows a representative example of Ru360 effects on PKA activity in the MM. Compared to control, 20 µM Ru360 did not affect PKA activity (0.99 ± 0.016%, Figure 4B). Following Ru360, 20 µM FSK did not alter PKA activity, while H-89 decreased the activity to 0.94 ± 0.01 (Figure 4B). Coadministration of Ru360 and FSK resulted in lower PKA activity compared to the maximal PKA activity achieved with FSK alone (Figure 4C) (*p* < 0.05). PKA activity caused by co-treatment with Ru360 and H-89 was similar (*p* = 0.42) to the minimal PKA activity caused by H-89 alone (Figure 4D). These findings suggest that mitochondrial Ca^2+^ indeed regulates PKA activity in the MM.

### 2.6. PKA Activity Is Affected by the Perturbation of the M Clock in hiPSC-CMs

**Figure 5:**
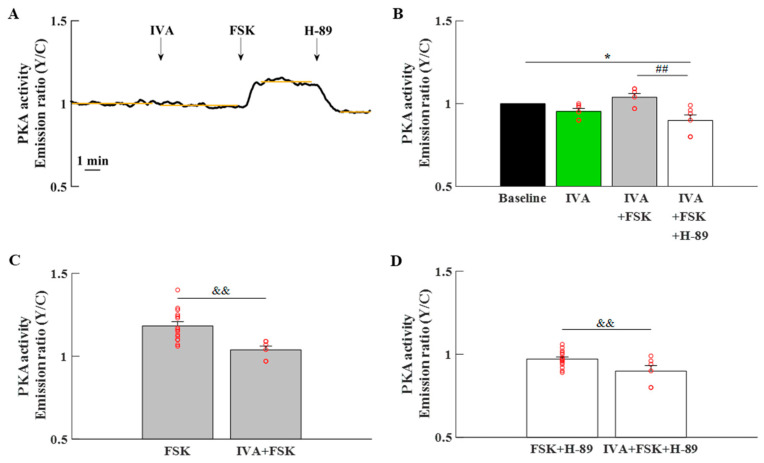
The effect of the If blocker ivabradine (IVA) on PKA activity in the cytosol of spontaneously beating hiPSC-CMs. (**A**) A representative example of cytosolic PKA activity in a single spontaneously beating hiPSC-CM in response to the I_f_ blocker ivabradine (IVA, 3 µM), followed by adenylyl cyclase (AC) activation with 20 µM forskolin (FSK) and PKA inhibition with 10 µM H-89. (**B**) The mean PKA activity in the cytosol in response to IVA, FSK, and H-89 (*n* = 6). (**C**) PKA activity in the cytosol of a single spontaneously beating hiPSC-CM following coadministration of IVA (3 µM) and FSK (20 µM). (**D**) PKA activity in the cytosol of a single spontaneously beating hiPSC-CM following coadministration of IVA (3 µM) and H-89 (10 µM). The data are presented as the mean ± SEM. * *p* < 0.05 IVA, FSK, or H-89 compared with a value of 1.0 using a one-sample *t*-test. ## *p* < 0.01 compared among IVA, FSK, and H-89 using a one-way ANOVA with Dunn–Šidák multiple comparisons. && *p* < 0.01 FSK or H-89 versus the response in the presence of IVA using an unpaired Student’s *t*-test.

To determine whether the M clock regulates PKA activity, hiPSC-CMs were treated with ivabradine (IVA), which blocks the pacemaker’s funny current (I_f_), indirectly affecting the Ca^2+^-clock and, thus, the degree of clock coupling [2]. As shown in Figure 5A, 3 µM IVA did not affect PKA activity in the cytosol. On average, following IVA, 20 µM FSK did not alter PKA activity, while H-89 decreased the activity to 0.89 ± 0.03. Coadministration of IVA and FSK resulted in lower PKA activity compared with the maximal PKA activity achieved with FSK alone (Figure 5C). Similarly, PKA activity caused by co-treatment with IVA and H-89 was lower (*p* < 0.05) than the minimal PKA activity caused by H-89 alone (Figure 5D). The reduction in the response to FSK when coadministered with IVA suggests that the degree of clock coupling affects PKA activity.

### 2.7. PKA Activity in the Cytosol Correlates with the Beating Rate of hiPSC-CMs

**Figure 6:**
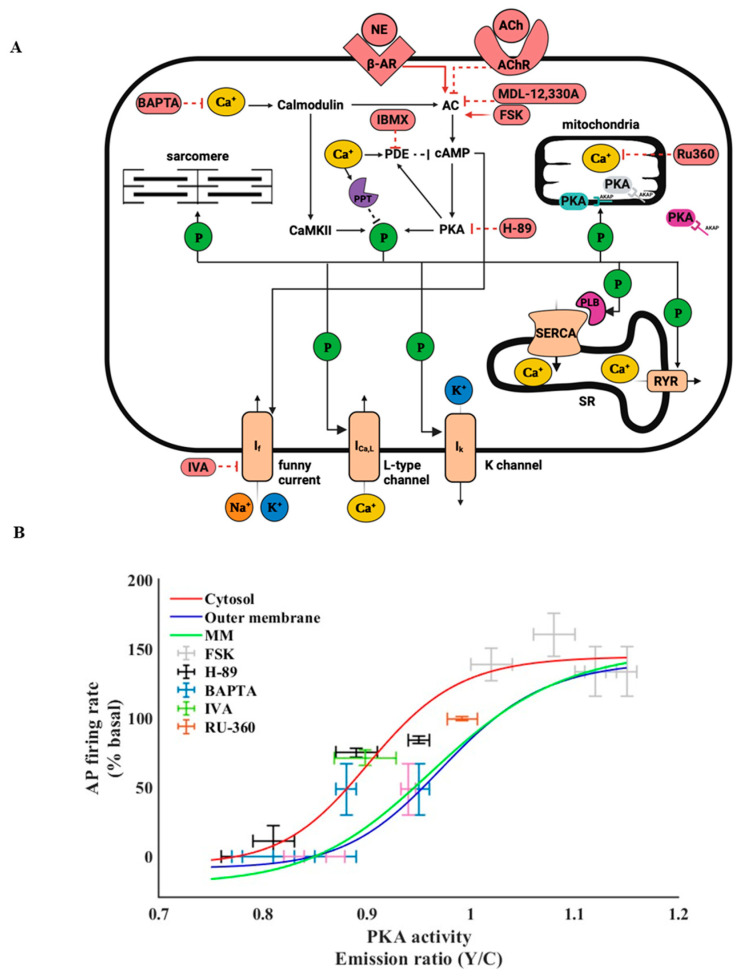
Spontaneous beating rate correlates with PKA activity in hiPSC-CMs. (**A**) A schematic model of pacemaker cell function. Ca^2+^ and membrane clocks are tightly coupled through Ca^2+^- and PKA-dependent activity in the cytosol and mitochondrial compartments. The model shows the effects of the tested drug on hiPSC-CM mechanisms. SERCA, sarcoplasmic reticulum Ca^2+^ ATPase; SR, sarcoplasmic reticulum; PLB, phospholamban; RyR, ryanodine; PDE, phosphodiesterase; PPT, protein phosphatase; *p*, phosphate; β-AR, adrenergic receptor; and ChR, cholinergic receptor. (**B**) Experimental measurement of PKA activity and spontaneous beating rate in hiPSC-CMs in response to AC activation with forskolin (FSK) (2, 10, and 20 µM; N = 6 and *n* = 10), PKA inhibition with H-89 (0.1, 1, 5, and 10 µM; *n* = 7 and *n* = 9), the Ca^2+^ chelator BAPTA (5, 10, and 25 µM; *n* = 6 and *n* = 12), and the I_f_ blocker ivabradine (IVA) (3 µM; N = 9 and *n* = 6), with N and n being the number of cells used for beating rate and PKA activity experiments, respectively. The equations for the curve fitting of the PKA-beating rate relationship in the cytosol: $-5.589+\frac{150\cdot x^{21.25}}{0.9044^{21.25}+x^{21.25}}$, outer mitochondrial membrane (OMM):$-8.612+\frac{150\cdot x^{20.17}}{0.9757^{20.17}+x^{20.17}},$ and mitochondrial matrix (MM): $-19.54+\frac{170\cdot x^{15.7}}{0.99686^{15.7}+x^{15.7}}$.

Here, we used a battery of drugs that affect different Ca^2+^-cAMP/PKA signaling pathways in hiPSC-CMs (marked in red, Figure 6A). To correlate hiPSC-CM function with PKA activity, the spontaneous beating rate and PKA activity before and after treatments with the above-mentioned drugs were plotted (Figure 6B). Figure 6B shows a tight sigmoidal correlation between the beating rate and PKA activity, along with sigmoidal correlations in the MM and OMM compartments.

### 2.8. PKA Compartmentalization in the SANC Cytosol, MM, and OMM

**Figure 7:**
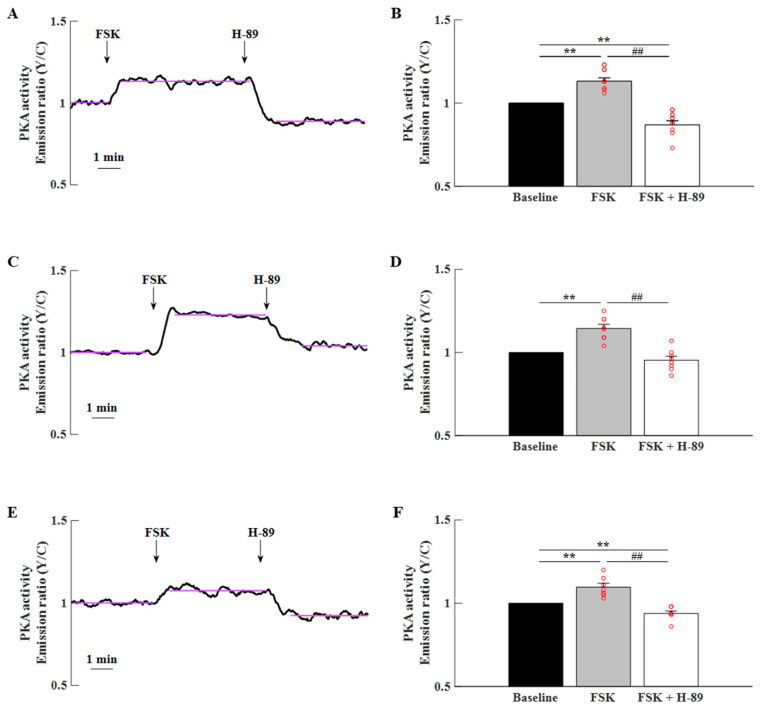
PKA activity in the cytosol and mitochondrial compartments of spontaneously beating SANCs. (**A**) A representative example of cytosolic PKA activity (AKAR4 emission Y/C) in a single SANC in response to adenylyl cyclase (AC) activation with 10 µM forskolin (FSK), followed by its inhibition with 10 µM H-89. (**B**) The mean PKA activity in the cytosol in response to FSK and H-89 (*n* = 8). (**C**) A representative example of PKA activity in the outer mitochondrial membrane (OMM, yTom-AKAR4 emission Y/C) in a single SANC in response to treatment with 10 µM FSK, followed with 10 µM H-89. (**D**) The mean PKA activity in the OMM in response to treatment with FSK and H-89 (*n* = 8). (**E**) A representative example of mitochondrial matrix (MM) PKA activity (4Cox8-AKAR4 emission Y/C) in a single SANC in response to treatment with 10 µM FSK, followed by 10 µM H-89. (**F**) The mean PKA activity in the MM in response to FSK and H-89 (*n* = 7). The data are presented as the mean ± SEM. ** *p* < 0.01 FSK or H-89 compared with a value of 1.0 using a one-sample *t*-test. ## *p* < 0.01 FSK versus H-89 using a paired Student’s *t*-test.

To compare the PKA regulation in hiPSC-CMs and SANCs, both cells were subjected to the treatments shown for hiPSC-CMs. To determine the relative PKA activity in the cytosol of SANCs, it was compared with the maximal and minimal activities. PKA activity in the SANC cytosol was increased by FSK (Figure S11A,B). A maximum effect was achieved by 10 µM FSK compared to the baseline. PKA activity was decreased by H-89 (Figure S12A,B), with the maximum effect obtained by 20 µM H-89. Because 10 µM H-89 provides a comparable effect to 20 µM H-89 on PKA activity, it was selected for further experiments. Characterization of basal PKA activity in the cytosol showed that it was increased by 10 µM FSK and decreased by 10 µM H-89 (Figure 7A). In summary, FSK increased PKA activity from 1.0 to 1.13 ± 0.02, and H-89 decreased the activity to 0.86 ± 0.02 (Figure 7B). Basal PKA activity in the OMM increased from 1.0 to 1.14 ± 0.02 with 10 µM FSK and decreased to 0.95 ± 0.02 by 10 µM H-89 (Figure 7C,D). Similar responses (1.09 ± 0.02 and 0.93 ± 0.01, respectively) were measured in the MM (Figure 7E,F). Taken together, the FSK-associated increase in PKA activity was similar (*p* = 0.347) in all compartments, while H-89 had a similar (*p* = 0.1157) effect in the cytosol and the MM, but a larger effect (*p* < 0.05) than in the OMM, suggesting that the effect of H-89 is compartment-dependent in SANCs. Basal PKA activity in the cytosol was 0.48 ± 0.06 when normalized to the activities measured in response to FSK and H-89. Similar (*p* = 0.4361) normalized values were documented in the MM (0.39 ± 0.08), but were lower (*p* < 0.05) in the OMM (0.21 ± 0.12).

### 2.9. Ca^2+^-Activated AC Is an Important Regulator of PKA Activity in SANCs

**Figure 8:**
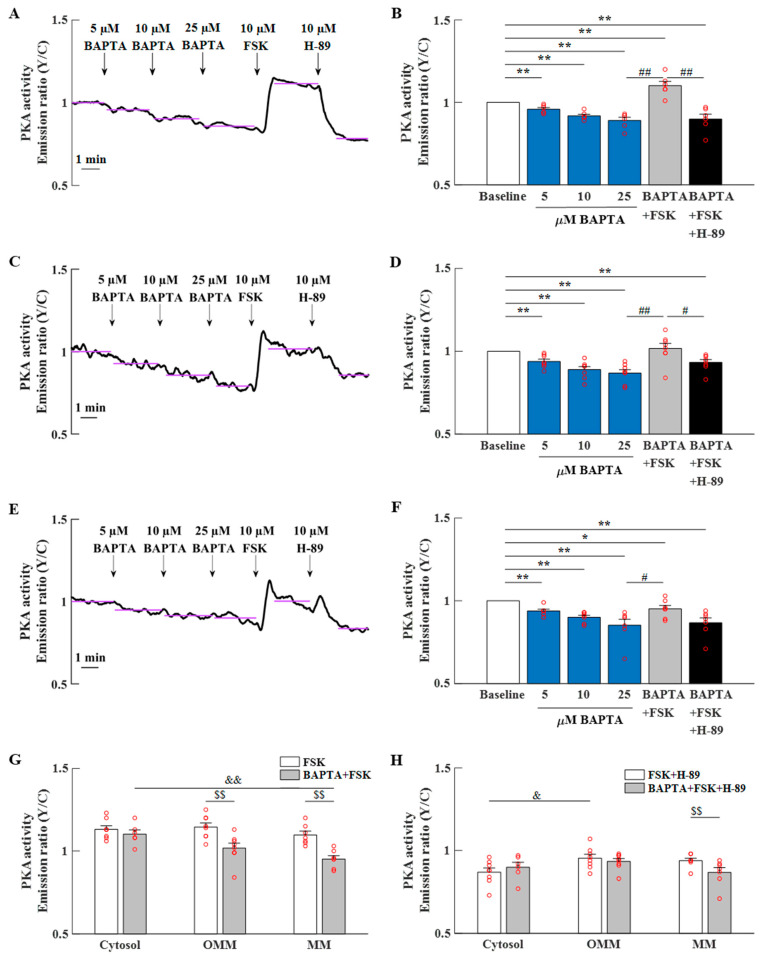
PKA activity in the cytosol and the mitochondrial compartments in single spontaneously beating SANCs. (**A**) A representative example of cytosolic PKA activity (AKAR4 emission Y/C) in a single pacemaker cell in response to treatment with increasing concentrations of a Ca^2+^ chelator BAPTA (5, 10, and 25 µM), followed by adenylyl cyclase (AC) activation using 10 µM forskolin (FSK) and PKA inhibition using 10 µM H-89. (**B**) The mean PKA activity in the cytosol in response to treatment with increasing concentrations of BAPTA, FSK, and H-89 (*n* = 6). (**C**) A representative example of PKA activity in the outer mitochondrial membrane (OMM, yTom-AKAR4 emission Y/C) in a single pacemaker cell in response to treatment with increasing concentrations of BAPTA (5, 10, and 25 µM), followed by treatment with 10 µM FSK and 10 µM H-89. (**D**) The mean PKA activity in the OMM in response to treatment with increasing concentrations of BAPTA, FSK, and H-89 (*n* = 8). (**E**) A representative example of mitochondrial matrix (MM) PKA activity (4Cox8-AKAR4 emission Y/C) in a single pacemaker cell in response to increasing concentrations of BAPTA (5, 10, and 25 µM), followed by 10 µM FSK and 10 µM H-89. (**F**) The mean PKA activity in the MM in response to treatment with increasing concentrations of BAPTA, FSK, and H-89 (*n* = 7). (**G**) PKA activity in the cytosol, OMM, and MM of SANCs following coadministration of BAPTA (25 µM) and FSK (10 µM). (**H**) PKA activity in the cytosol, OMM, and MM of SANCs following coadministration of BAPTA (25 µM) and H-89 (10 µM). The data are presented as the mean± SEM. * *p* < 0.05 and ** *p* < 0.01BAPTA, FSK, or H-89 compared with a value of 1.0 using a one-sample *t*-test. # *p* < 0.05 and ## *p* < 0.01 compared among BAPTA, FSK, and H-89 using a one-way ANOVA with Dunn–Šidák multiple comparison. $$ *p* < 0.01 FSK or H-89 versus the response in the presence of BAPTA in the same compartment using an unpaired Student’s *t*-test. & *p* < 0.05 and && *p* < 0.01 compared among FSK or H-89 responses in the cytosol, OMM, and MM using a one-way ANOVA with Dunn–Šidák multiple comparison. The line presents the response to each drug where the mean of the sliding window was within 5% of the average value after the transition period.

Next, to determine whether Ca^2+^ regulates PKA activity in SANCs like in hiPSC-CMs, SANCs were treated with increasing concentrations of BAPTA. Compared to control, 25 µM BAPTA reduced PKA activity by 11 ± 1.89% in the cytosol (Figure 8A,B), by 13.1 ± 2.02% in the OMM (Figure 8C,D), and by 14.71 ± 3.62% in the MM (Figure 8E,F). These findings suggest that like in hiPSC-CMs, in SANCs Ca^2+^ is involved in regulating PKA activity in different cellular compartments.

To determine whether a reduced Ca^2+^ concentration limits the maximal PKA activity generated by AC activation, maximal PKA activity was induced by 10 µM FSK following BAPTA treatment. The dual treatment resulted in a similar (*p* = 0.3823) PKA activity in the cytosol (1.1 ± 0.02 vs. 1.13 ± 0.02 in the FSK control). In contrast, this treatment reduced (*p* < 0.0015) PKA activity in the OMM (1.01 ± 0.03 vs. 1.14 ± 0.02 when only FSK was used) and in the MM (0.95 ± 0.02 vs. 1.09 ± 0.02 in the FSK control) compared to the maximal PKA activity caused by FSK only, in either compartment (Figure 8G). Coadministration of BAPTA and FSK resulted in similar (*p* = 0.1123) PKA activity in the OMM compared to wthe cytosol, but with lower PKA activity in the MM only in the absence of Ca^2+^, suggesting that Ca^2+^ indeed regulates PKA activity and affects PKA compartmentation. Coadministration of BAPTA and 10 µM H-89 resulted in similar (*p* = 0.5) PKA activity in the cytosol (0.89 ± 0.02 vs. 0.86 ± 0.02 only H-89) (Figure 8H) and in the OMM (0.93 ± 0.01 vs. 0.95 ± 0.02 only H-89) (Figure 8H), but to reduced (*p* < 0.05) PKA activity in the MM (0.86 ± 0.02 vs. 0.93 ± 0.01 in the H-89 control) compared to the minimal PKA activity achieved by adding only H-89 (Figure 8H). Coadministration of BAPTA and H-89 resulted in similar (*p* = 0.1818) PKA activity in the MM and OMM compared to the cytosol. Note that when H-89 was applied alone without BAPTA, PKA activity was different in the MM and OMM.

## 3. Discussion

In the present study, we investigated the spatiotemporal PKA dynamics in spontaneously beating hiPSC-CMs and SANCs. The major findings are: (1) The inhibition of Ca^2+^-activated, PKA-dependent signaling led to an imbalance between ATP supply and demand, supporting the first hypothesis that Ca^2+^-activated, PKA-dependent signaling is an important regulator of energy balance. (2) The PKA inhibitor H-89 caused distinct compartmentalization of PKA activity, supporting the first hypothesis that PKA dynamics are different in the cytosol than in the mitochondrial compartments. (3) AC1 and AC8 were detected in hiPSC-CMs, and pharmacological modulation of their activity (Figure 6B) demonstrated a sigmoidal correlation between the spontaneous beating rate and PKA activity, supporting the second hypothesis that the function of spontaneously beating hiPSC-CMs correlates with the activity of the Ca^2+^-activated ACs. (4) In the presence of the Ca^2+^ chelator BAPTA, the compartmentalization effect of H-89 was diminished, supporting the third hypothesis that PKA compartmentalization is regulated by Ca^2+^. (5) PKA dynamics and their Ca^2+^-dependent activity were similar in rabbit SANCs and hiPSC-CMs.

### 3.1. Ca^2+^-Activated, PKA-Dependent Signaling Is an Important Regulator of Energy Balance

This is the first study to investigate the regulators of ATP supply–demand balance in hiPSC-CMs. PKA and Ca^2+^ signaling regulate major ATP consumers in the cell, including the SR pump, myofilament contraction, and the Na^+^-K^+^ pump. A reduction in ATP supply affects hiPSC-CM function and, specifically, the spontaneous beating ability of hiPSC-CMs. Mitochondrial PKA also controls energetic balance [17] via phosphorylation of complexes I, IV, and V in the electron transport chain [23], by controlling the rate of ATP delivery from the mitochondria to the cytosol through the ANT [24], and by phosphorylation of the mitochondrial Na^+^/Ca^2+^ exchanger [25]. The phosphorylation sites in the complexes are located both in the inner membrane and matrix. Different mitochondrial PKA activity in the OMM and MM may be related to a difference in function. In SANCs under basal conditions, PKA plays a key role in matching ATP supply to demand [17]. At higher energy demands, both mitochondrial Ca^2+^ and PKA signaling become critical regulators [26]. Similarly, in hiPSC-CMs, both mitochondrial Ca^2+^ and PKA signaling serve as essential mediators of energy balance, even under basal conditions.

### 3.2. High PKA Activity in hiPSC-CMs Is Distributed Differently in Mitochondrial vs. Cytosolic Compartments

This is the first study to measure PKA activity in specific hiPSC-CMs intracellular compartments. Cytosolic cAMP activity was previously measured in hiPSC-CMs [27], with maximal activity caused by FSK. A similar maximal drug response was obtained here for PKA activity. H-89 was shown to decrease spontaneous beating rate in rabbit SANCs [12] and PKA activity. These results are in agreement with the correlation of action potential beating rate and PKA activity reported in rabbit SANCs [28]. In agreement with the responses observed in rabbit SANCs, minimal PKA activity was achieved by H-89. Scaling PKA activity to its maximal and minimal values showed that high PKA activity is present in hiPSC-CMs under basal conditions in both cytosolic and mitochondrial compartments, with higher activity in the mitochondrial compartments than in the cytosolic compartments.

### 3.3. The Role of cAMP/PKA Activity in hiPSC-CMs

PKA controls the Ca^2+^ clock by affecting the rate of Ca^2+^ release and reuptake into the SR [12]. PKA also controls the M clock by affecting the inactivation kinetics of L-type Ca^2+^ current (I_Ca,L_) and the delayed rectifier K^+^ current (I_k_) [29]. Thus, both cAMP and PKA signaling have markedly impacted the spontaneous beating rate. The sigmoidal relationship between the spontaneous beating rate and PKA activity (Figure 6B) further supports the hypothesis regarding the role of PKA in hiPSC-CMs function. Although cAMP controls I_f_, blocking the current by IVA did not reduce PKA activity; however, application of FSK or H-89 combined with IVA reduced PKA activity compared to FSK or H-89 alone. At the tested concentration, IVA is a direct inhibitor of I_f_ [30]. However, as previously shown in SANCs [3] and hiPSC-CMs [2], IVA also indirectly alters Ca^2+^ release, which in turn affects the beating rate. Thus, the reduced degree of clock coupling caused by IVA shifts the beat interval and, consequently, the set point of PKA activity. This may explain why PKA activity is affected when I_f_ is reduced. Because the clocks are coupled, it is impossible to deactivate the Ca^2+^ clock and test the M clock separately.

### 3.4. PKA Activity and Its Compartmentalization Are Affected by Ca^2+^

The Ca^2+^ chelator BAPTA reduced PKA activity similarly in the cytosol and mitochondrial compartments (Figure 3). Comparing our results with the previous finding in mice SANCs suggests that Ca^2+^-activated AC1 and AC8 are also located in the mitochondria [15], and that Ca^2+^-dependent PDE and PPT are present in hiPSC-CMs mitochondria. PDE4 [31], PDE1C, PDE3A, and PDE5A [27] were shown to be the dominant PDE types in hiPSC-CMs. Thus, Ca^2+^ activates ACs which increase PKA activity, while it may also limit PKA activity by regulating PKA degradation. Additionally, PDE4 and PDE5A activities are regulated by cAMP [32], and therefore increased cAMP levels increase PKA activity, while also limiting it by regulating PKA degradation. In the presence of a Ca^2+^ chelator, the compartmentalization effect of the PKA inhibitor H-89 is diminished, while H-89 reduces the degree of coupling between the two clocks [13]. It is likely that loss of compartmentalization may be partially responsible for reduced pacemaker activity in the presence of a Ca^2+^ chelator. Forskolin increased PKA activity in the cytosol even after BAPTA treatment, suggesting the presence of additional AC isoforms that are not Ca^2+^-activated. Indeed, AC3, AC4, AC5, AC6, AC7 and AC9 were identified in hiPSC-CMs [16]. Similarly, in rabbit SANCs, AC2, AC5 and AC6 were also expressed alongside the Ca^2+^-activated isoforms AC1 and AC8 [13].

The molecular mechanisms underlying the Ca^2+^-dependent compartmentalization of PKA signaling in hiPSC-CMs remain to be elucidated. A likely mechanism involves AKAP family scaffolding proteins that spatially restrict PKA activity within mitochondrial and cytosolic microdomains. In particular, the mitochondrial scaffold AKAP1 represents a strong candidate for organizing OMM-associated PKA signaling, although the determinants of matrix-localized PKA activity remain unclear. Future studies combining AKAP perturbation strategies with compartment-specific biosensors are required to define the scaffolds responsible for Ca^2+^-dependent redistribution of PKA signaling.

The observed Ca^2+^-dependent compartmentalization of PKA signaling may have important implications for the use of hiPSC-CMs in cardiac disease modeling and regenerative medicine. Because pathological remodeling of Ca^2+^ handling and β-adrenergic signaling are central to arrhythmias and heart failure, compartment-specific PKA dynamics could provide a sensitive functional readout of disease-associated signaling defects. In addition, the spatial organization of mitochondrial and cytosolic PKA signaling may reflect the metabolic and electrophysiological maturation state of hiPSC-CMs, with potential relevance for optimizing their therapeutic utility in cell replacement strategies.

### 3.5. PKA Dynamics and Their Ca^2+^-Dependent Activity Are Similar in Spontaneously Beating hiPSC-CMs and Rabbit SANCs

Like in hiPSC-CMs, Ca^2+^-mediated PKA compartmentalization is present in SANCs. This result suggests that the SANCs coupled-clock system has a specific regulator in different compartments, and that all the compartments are orchestrated to control the degree of coupling of the coupled-clock system. In rabbit SANCs, PKA activity is higher than in mouse ventricular cells [33]. Interestingly, not only is PKA synthesis high in SANCs but because PDE activity that degrades PKA is high, the rate of PKA synthesis is faster than its degradation. In the present work, we employed rabbit SANCs because they express AC1 and AC8, and their Ca^2+^ transient is more similar to humans than to murine SANCs [34]. The fact that distinct responses to BAPTA and FSK occurred in SANCs compared to hiPSC-CMs may relate to the higher spontaneous basal beating rate of SANCs (~2.5 Hz) compared to hiPSC-CMs (1 Hz).

The differences in compartment-specific PKA dynamics observed between hiPSC-CMs and SANCs may also reflect the relatively immature structural and signaling organization of hiPSC-CMs. While these findings highlight limitations in the ability of hiPSC-CMs to fully recapitulate adult pacemaker cell physiology, particularly with respect to highly localized Ca^2+^-dependent signaling microdomains, they also demonstrate that essential features of compartmentalized PKA signaling are retained. Accordingly, hiPSC-CMs remain a useful platform for investigating human cardiac signaling mechanisms, while also providing a framework for evaluating strategies to enhance cardiomyocyte maturation and pacemaker-like phenotypes.

### 3.6. Study Limitations

Although pure iPSC-derived SAN hiPSC-CMs [35] were not used in this study, Ca^2+^-dependent PKA dynamics are comparable between hiPSC-CMs and rabbit SANCs. It was previously shown that action potential characteristics [36], coupled-clock mechanisms [2], and M clock components [37] are comparable between human SANCs and spontaneously beating hiPSC-CMs.

Note that although sensitivity to photobleaching is similar between compartments (Figure S8), pH which may vary across compartments, could affect the biosensors differently, an effect that could be further complicated by differences in biosensor concentration. Comparing the intensity of probes across different compartments reflected differences in probe efficiency rather than true biological variation. Therefore, we normalized the activity for each probe individually.

A limitation of this study is the use of 30–60-day-old hiPSC-CMs, which are known to retain features of structural and electrophysiological immaturity relative to adult cardiomyocytes and SANCs. Immature hiPSC-CMs may exhibit altered sarcomeric organization, ion channel expression, Ca^2+^ handling, and metabolic profiles, all of which could influence cAMP/PKA signaling dynamics and compartmentalization. Although the cells used in this study displayed robust spontaneous activity and reproducible PKA-dependent responses, we did not perform a comprehensive maturation analysis or directly compared signaling properties across different maturation stages. Therefore, it remains possible that the spatial organization and functional regulation of PKA signaling observed here differ quantitatively or qualitatively from those in fully mature human cardiomyocytes or primary SANCs. Future studies incorporating prolonged culture, engineered maturation approaches, or adult human cardiac tissue models will be important to determine the extent to which developmental maturity influences PKA compartmentalization and its physiological relevance.

## 4. Materials and Methods

### 4.1. hiPSC Generation and Differentiation into Cardiomyocytes

hiPSCs were generated from a skin biopsy obtained from a 42-year-old healthy female (clone 24.5) and from a foreskin obtained from a healthy neonate (clone FSE-5m), as previously described [38,39].The cell lines used in this study were obtained from the Ofer Binah Laboratory. hiPSCs were differentiated into hiPSC-CMs, according to the directed differentiation protocol, by modulating Wnt/β-catenin signaling, as previously described [40]. Experiments were performed on 30–60-day-old hiPSC-CMs replated as single cells on experimental dishes, as previously described [2], in Tyrode’s solution (at 37 °C) containing (in mM): 140 NaCl, 5.4 KCl, 10 HEPES, 2 Na-pyruvate, 10 glucose, 1 MgCl_2_, and 2 CaCl_2_, with pH 7 adjusted using NaOH.

### 4.2. Isolation of Rabbit SANCs

The animals were treated in accordance with the Technion Ethics Committee guidelines. The experimental protocols were approved by the Technion Animal Care and Use Committee (approval number IL-001-01-19). Single, spindle-shaped, spontaneously beating SANCs were isolated from New Zealand white rabbit hearts, as previously described [41], and cultured, as previously described [20]. Experiments were performed on SANCs in HEPES solution (at 37 °C) containing (in mM): 140 NaCl, 5.4 KCl, 2 MgCl_2_, 5 HEPES, 1.8 CaCl_2_, and 5.5 glucose, with pH 7.4 adjusted using NaOH.

### 4.3. Measurement of PKA Dynamics

hiPSC-CMs and SANCs were infected with adenoviral particles carrying vectors expressing cytosolic, OMM, or MM PKA probes: pcDNA3-A-kinase activity receptor 4 (pcDNA3-AKAR4, Addgene, Watertown, MA, USA), yTom-AKAR4, and 4Cox8-AKAR4, respectively. The OMM and MM plasmids were kind gifts from Aldebaran M. Hofer (Harvard Medical School, West Roxbury, MA, USA). Plasmids containing the FRET sensor were generated by Vector Biolabs (Malven, PA, USA). The cytosolic, OMM and MM PKA probes were diluted to 1:200,000, 1:500,000, or 1:250,000, respectively, in culture medium, or to 1:133,300, 1:222,200, or 1:117,600 dilution in serum-free culture medium, for hiPSC-CMs and SANC infection, respectively. Twenty-four h after infection, before cell imaging, the culture medium was aspirated, and the dishes were washed several times with cell-type Tyrode’s/HEPES solution. Real-time FRET imaging experiments were performed on an LSM880 confocal microscope (Zeiss, Oberkochen, Germany) at 37 ± 0.5 °C, under a 40×/1.2 N.A. water immersion lens. The cells were excited at a wavelength of 405 nm, and cyan fluorescent protein (CFP) and yellow fluorescent protein (YFP) fluorescence emissions were measured at wavelengths of 470–512 nm and 517–585 nm, respectively, with a 458/514 nm mirror beam splitter. The fluorescence images were analyzed using a custom MATLAB R2026a program. Briefly, the images were background-corrected by subtracting the fluorescence intensity of the background without cells from the emission intensities of cells expressing fluorescent reporters. The YFP/CFP intensity ratio of the entire signal was normalized by dividing each value by the average basal value measured before addition of stimulant/inhibitor. The response to each drug was calculated at steady state, comparing the value of a 10 s sliding window lowpass filter with a threshold taken as the mean value of the segment starting 30 s after the beginning of the segment (this part of the segment was considered transient-free). If the mean of the sliding window was within 5% of the threshold, the transient period was considered to be over, and the rest of the segment was used, excluding the values above 5%. Note that no significant decay in fluorescence was observed over 20 min for hiPSC-CMs (Figure S13) and SANCs (Figure S14) in neither compartment.

### 4.4. Staining of Outer Mitochondrial Membrane and Mitochondrial Matrix

To determine the localization of 4Cox8-AKAR4 in the MM or yTom-AKAR4 in the OMM, cells were infected with PKA probes and incubated for 24 h. Thereafter, hiPSC-CMs were loaded with 2 nM MitoTracker red (an indicator of MM, Invitrogen, Waltham, MA, USA) or 5 nM MitoTracker green (an indicator of OMM, Invitrogen) for 15 or 30 min, respectively, at room temperature (RT) RT. Rabbit SANCs were loaded with 10 nM MitoTracker red or 25 nM MitoTracker green for 15 or 30 min, respectively. Cells were washed with Tyrode’s (hiPSC-CMs) or HEPES (SANCs) solutions and imaged with a LSM880 confocal microscope (Zeiss) under a 63×/1.4 N.A. oil immersion objective at 37 ± 0.5 °C. The CFP and both Mito Trackers were excited simultaneously by the 405 nm and 488 nm lasers, respectively. For the CFP and MitoTracker red imaging in hiPSC-CMs and SANCs, the CFP emission was measured at wavelengths of 470–512 nm, and MitoTracker red was measured at 550–700 nm. For the CFP and MitoTracker green imaging in hiPSC-CMs and SANCs, the CFP emission was measured at wavelengths of 460–480 nm, and MitoTracker green was measured at 500–600 nm. Figure S15A, showing a confocal image of hiPSC-CMs expressing 4Cox8-AKAR4 and loaded with MitoTracker red, demonstrates localization of 4Cox8-AKAR4. Figure S15B, showing a confocal image of a hiPSC-CM expressing yTom-AKAR4 and loaded with MitoTracker green, illustrates localization of yTom-AKAR4. Figure S16 shows SANCs expressing the above probes and loaded with the fluorescent OMM and MM indicators.

To further determine the localization of 4Cox8-AKAR4 in the MM or yTom-AKAR4 in the OMM, cells were infected with PKA probes and incubated for 24 h. hiPSC-CMs infected with AKAR4 were permeabilized on the microscope stage using 5 µM digitonin in the presence of 10 µM cAMP. Figure S17A shows that in these hiPSC-CMs, the fluorescence diminished. In the presence of 25 µM digitonin and 10 µM cAMP in the media, we were able to precisely detect the moment at which the plasma membrane became permeabilized in individual cells, as measured by the activation of yTom-AKAR4 (increase in fluorescence, Figure S17B) vs. 4Cox8-AKAR4 (no change in fluorescence) (Figure S17C). The data shown are from one representative experiment out of three repeats.

### 4.5. Measuring the Spontaneous Beating Rate and Ca^2+^ Characteristics of hiPSC-CMs

Ca^2+^ cycling in single hiPSC-CMs was recorded by monitoring the fluorescence of the Ca^2+^ indicator dye Fluo-4 AM (Thermo Fisher Scientific, Waltham, MA, USA), using a LSM880 confocal microscope (Zeiss, Oberkochen, Germany), as previously described [2]. hiPSC-CMs were loaded with 2.5 μM Fluo-4 AM for 20 min in the dark at room temperature (RT), and then washed with Tyrode’s solution. Fluorescence was observed by exciting the sample with a 488 nm argon laser and measuring fluorescence emission with LP 505 nm. Ca^2+^ images were acquired using a 40×/1.2 water immersion lens with a line-scan mode (1.22 ms per scan; pixel size, 0.01 µm). The beating rate and other Ca^2+^ characteristics were calculated by analyzing line-scan images using a modified version of a semi-automatic Matlab Graphic User Interface named Sparkalyzer [41].

### 4.6. AC1 and AC8 Staining in hiPSC-CMs

AC subtypes were detected using immunostaining and confocal microscopy, as previously described [14]. Briefly, hiPSC-CMs were first fixed with 4% paraformaldehyde (Thermo Fisher Scientific, Waltham, MA, USA) in phosphate-buffered saline (PBS) (Gibco, Gaithersburg, Maryland, USA) for 15 min at RT, and then rinsed with PBS (3 changes, 10 min each). Next, hiPSC-CMs were permeabilized with Triton X-100 (Sigma-Aldrich, Burlington, MA, USA) (0.1% in PBS) for 15 min at RT, and then washed in PBS (3 changes, 10 min each). Subsequently, the cells were blocked with 10% normal donkey serum (Jackson ImmunoResearch, Cambridge, UK) in PBS for 60 min at RT, followed by incubation with primary antibodies raised against AC1 and AC8 (Proteintech, Planegg-Martinsried, Germany, 1:200 dilution in PBS for both primary antibodies) at 4 °C overnight. The cells were again washed in PBS (3 changes, 10 min each) and incubated with the secondary antibody (Alexa Fluor 488-conjugated goat anti-rabbit (Invitrogen, Waltham, MA, USA), 1:400 dilution in PBS) at RT for 60 min, before being washed again with PBS (3 changes, 10 min each). The cells were stored in the dark at 4 °C and imaged within 2 days using a LSM880 confocal microscope (Zeiss) under a 63×/1.4 N.A. oil immersion objective. For the control experiments, the cells were not incubated with the primary antibody.

### 4.7. Flavoprotein Autofluorescence Recordings

The endogenous autofluorescence of mitochondrial flavoproteins was imaged in Tyrode’s solution at 37 ± 0.5 °C using a LSM880. Fluorescence was observed by exciting the sample with a 488 nm argon laser. Autofluorescence images were acquired using a 40×/1.2 water immersion lens. The flavoprotein fluorescence response (an indicator of matrix redox state) to each drug in a given cell was expressed as a percent change from the baseline.

### 4.8. Drugs

The AC activator FSK, the PKA inhibitor H-89, the PDE inhibitor IBMX, the cell-permeable and irreversible inhibitor adenylate cyclase (MDL-12,330A, Hydrochloride), the blocker of mitochondrial Ca^2+^ uptake into (Ru360), the selective inhibitor of Ca^2+^–calmodulin-dependent phosphodiesterase I (PDE1), 8-Methoxymethyl-3-isobutyl-1-methylxanthine (MIMX), the protein phosphatase 2A (PP2A) and PP1 inhibitor calyculin A (CyA), and the PP2A Okadaic acid (OA) inhibitor were purchased from Sigma-Aldrich. The cytosolic Ca^2+^ chelator BAPTA-AM was purchased from ENCO (Cayman Chemical, Ann Arbor, MI, USA). The pacemaker current I_f_ blocker IVA was purchased from Toronto Research Chemicals (North York, ON, Canada).

### 4.9. Statistics

The data were calculated as the mean ± SEM. *n* represents the number of rabbits or hiPSC-CM clusters. The responses to each drug were compared with a basal fixed value of 1.0 using a one-sample *t*-test. The bar graphs include the presentation of the basal mean PKA activity of 1.0 solely for visual comparison purposes. When two drugs were tested, the data were compared using a paired or unpaired Student’s *t*-test to differentiate between the two drugs. When more than two drugs were tested, data were compared using one-way ANOVA with the Dunn–Šidák multiple comparison to differentiate between the conditions. The statistical method used in each case is indicated in the respective figure legend. Differences were considered statistically significant at *p* < 0.05 or *p* < 0.01, as indicated in each figure. All data are obtained from at least three independent hiPSC-CM differentiations and four rabbits.

## 5. Conclusions

PKA activity controls and is controlled by the membrane and Ca^2+^ clocks. PKA activity is differentially compartmentalized in the MM and OMM compared with the cytosol, and Ca^2+^ plays a key role in the compartmentalization of PKA activity.

## Data Availability

The data supporting reported results can be requested from Prof. Yael Yaniv at yaely@bm.technion.ac.il.

## Supplementary Materials

The following supporting information can be downloaded at: https://www.mdpi.com/article/10.3390/ijms27156559/s1.

## Abbreviations

The following abbreviations are used in this manuscript:

hiPSC-CMs	Human-induced Pluripotent Stem Cell-derived cardiomyocytes
LCRs	Local Ca^2+^ releases
cAMP	Cyclic adenosine monophosphate
PKA	Protein kinase A
SANCs	Sinoatrial node cells
M	Membrane
SR	Sarcoplasmic reticulum
AC	Adenylyl cyclase
FRET	Fluorescence resonance energy transfer
OMM	Outer mitochondrial membrane
MM	Mitochondrial matrix
CFP	Cyan fluorescent protein
RT	Room temperature
FSK	Forskolin
PDE	Phosphodiesterase
IBMX	Inhibitor 3-isobutyl-1-methylxanthine
IVA	Ivabradine
MDL	MDL-12,330A
I_f_	Funny current
