# Supplementary material for: Intracellular Ca2+ Modulates PKA Compartmentalization and Dynamics in Human iPSC-Derived Cardiomyocytes
Source: Int J Mol Sci. 2026 Jul 23;27(15):6559. doi: 10.3390/ijms27156559 (PMC13466060; doi:10.3390/ijms27156559)
Supplement: Supplementary file 1 [file ijms-27-06559-s001.zip › ijms-4097681-supplementary.pdf]

Supplement

Intracellular Ca^{2+} Modulates PKA Compartmentalization and Dynamics in Human iPSC-Derived Cardiomyocytes

**Anat Rotschield [1,†], Savyon Mazgaoker [1,†], Sofia Segal [1], Ido Weiser-Bitoun [1,2,3], Inbar Brosh [1], Ofer Binah [4]
and Yael Yaniv [1,*]**

[1] Laboratory of Bioelectric and Bioenergetic Systems, Faculty of Biomedical Engineering, Technion-IIT,
Haifa 3200003, Israel; anat.ro@campus.technion.ac.il (A.R.); broshi@gmail.com (I.B.)
[2] Department of Internal Medicine "C", Rambam Health Care Campus, Haifa 3109601, Israel
[3] The Leir Clinical Research Institute at Rambam (CRIR), Rambam Health Care Campus, Haifa, Israel
[4] Department of Physiology, Biophysics and Systems Biology, Ruth and Bruce Rappaport Faculty of Medicine and Research Institute, Technion-IIT, Haifa 3525433, Israel; binah@technion.ac.il
* Correspondence: yaely@bm.technion.ac.il; Tel.: +972-4-8294124
† These authors contributed equally to this work.

Abstract

The automaticity of human-induced Pluripotent Stem Cell-derived cardiomyocytes (hiPSC-CMs) is governed by coupled Ca^{2+} and membrane clocks, coordinated through local Ca^{2+} releases (LCRs) and cyclic adenosine monophosphate (cAMP)/protein kinase A (PKA) signaling. We investigated the role of PKA in hiPSC-CM energetics by measuring its dynamics in the cytosol and mitochondria, and its crosstalk with Ca^{2+}. We tested three hypotheses: (i) Ca^{2+}-activated PKA signaling regulates energy balance; (ii) adenylyl cyclase activity correlates with spontaneous beating; and (iii) PKA compartmentalization is Ca^{2+}-dependent. We also compared hiPSC-CMs with rabbit sinoatrial node cells (SANCs). The key findings are: (i) PKA inhibition (H-89), Ca^{2+} chelation (BAPTA), or mitochondrial Ca^{2+} blockade (Ru360) led to energy imbalance; (ii) H-89 induced compartmentalized PKA activity in the cytosol, mitochondrial matrix, and outer mitochondrial membrane; (iii) Ca^{2+} chelation with BAPTA reduced PKA activity globally; and (iv) PKA dynamics and Ca^{2+}-dependent regulation were similar in hiPSC-CMs and rabbit SANCs. In conclusion, intracellular Ca^{2+}-mediated PKA compartmentalization is present in hiPSC-CMs and rabbit SANCs.

Keywords: cyclic adenosine monophosphate (cAMP); protein kinase A (PKA); pacemaker; PKA compartmentalization; hiPSC-CMs; sinoatrial node cells (SANCs)

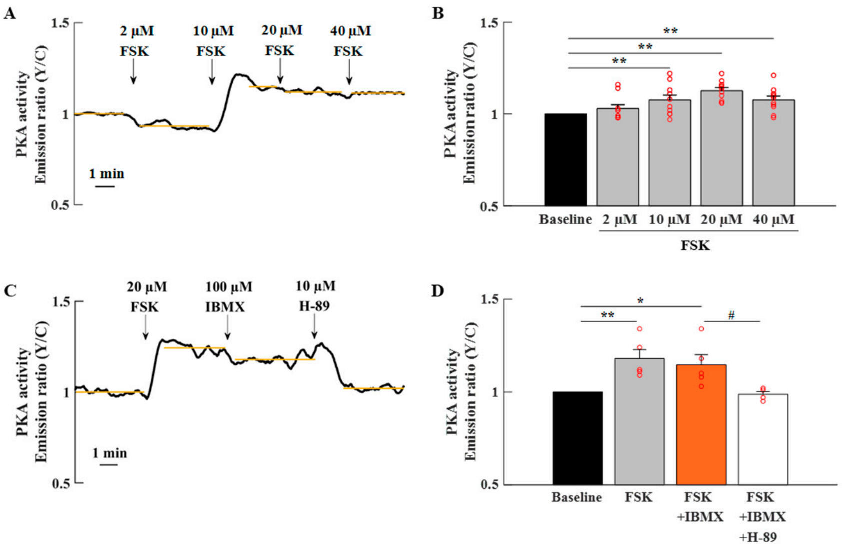

Figure S1. The effect of the adenylyl cyclase (AC) activator (forskolin) on PKA activity in the cytosol of spontaneously beating hiPSC-CMs. (**A**) A representative example of cytosolic PKA activity (AKAR4 emission Y/C) in a single hiPSC-CM in response to increasing concentrations of forskolin (FSK) (2, 10, 20, and 40 μM). (**B**) The mean PKA activity in the cytosol in response to increasing concentrations of FSK (n = 10). (**C**) A representative example of cytosolic PKA activity in a single hiPSC-CM in response to 20 μM FSK, followed by addition of the phosphodiesterase inhibitor IBMX (100 μM) and 10 μM H-89. (**D**). The mean PKA activity in the cytosol in response to FSK, IBMX, and H-89 (n = 5). The data are presented as the mean ± SEM. * $p < 0.05$ and ** $p < 0.01$FSK, IBMX, or H-89 compared with a value of 1.0 using a one-sample t-test. # $p < 0.05$ compared among FSK, IBMX, and H-89 using a one-way ANOVA with Dunn–Šidák multiple comparison.

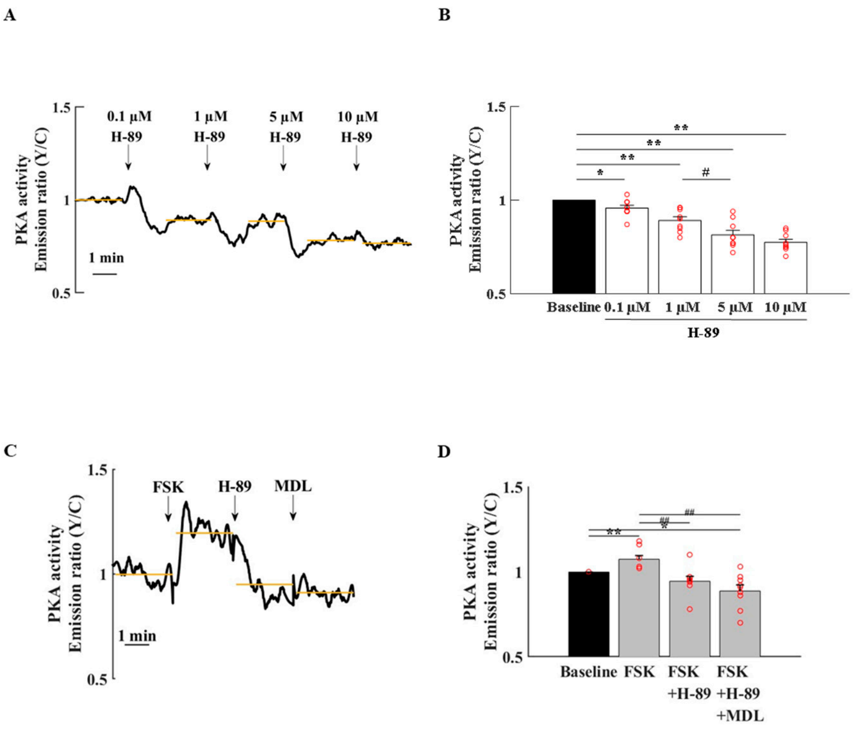

Figure S2. The effect of PKA inhibition (H-89) on PKA activity in the cytosol of spontaneously beating hiPSC-CMs. (**A**) A representative example of cytosolic PKA activity (AKAR4 emission Y/C) in a single hiPSC-CM in response to treatment with increasing concentrations of the PKA inhibitor H-89 (0.1, 1, 5, and 10μM). (**B**) The mean PKA activity in the cytosol in response to increasing concentrations of H-89 (*n* = 9). (**C**) A representative example of cytosolic PKA activity (AKAR4 emission Y/C) in a single hiPSC-CM in response to treatment with FSK, followed by the PKA inhibitor H-89 (10μM) and MDL (400μM). (**D**) The mean PKA activity in the cytosol in response to FSK, followed by the PKA inhibitor H-89 and MDL (*n* = 9). The data are presented as the mean ± SEM. * $p < 0.05$ and ** $p < 0.01$ H-89 responses compared with a value of 1.0 using a one-sample *t*-test. ## $p < 0.05$ and ## $p < 0.01$ compared among H-89 responses using a one-way ANOVA with Dunn–Šidák multiple comparison.

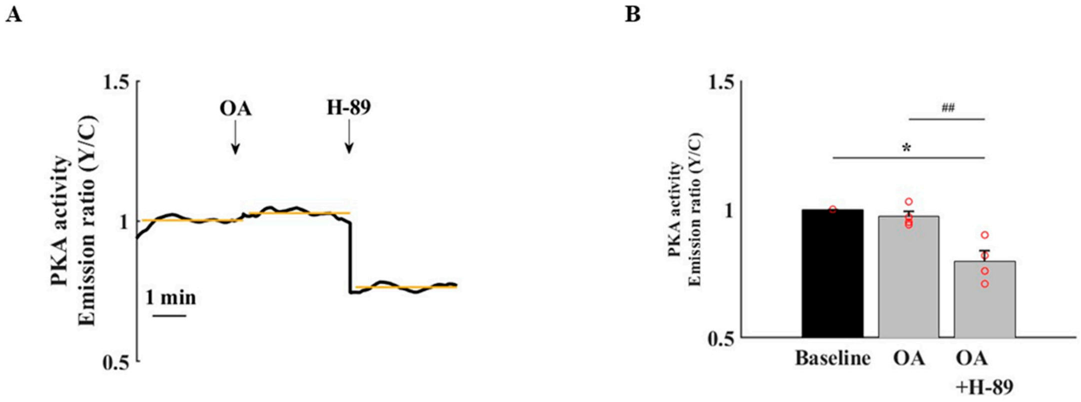

Figure S3. The effect of the PP2A inhibitor okadaic acid (OA) on PKA activity in the cytosol compartment of spontaneously beating hiPSC-CMs. (**A**) A representative example of cytosol compartment PKA activity in a single spontaneously beating hiPSC-CM in response to the PP2A inhibitor okadaic acid (OA) (100 μM), followed by PKA inhibition with 10 μM H-89. (**B**) The mean PKA activity in the cytosol compartment in a single spontaneously beating hiPSC-CM in response to the PP2A inhibitor okadaic acid (OA) (100 μM), followed by PKA inhibition with 10 μM H-89 (*n* = 4). The data are presented as the mean ± SEM. * $p < 0.05$ OA or H-89 compared with a value of 1.0 using a one-sample *t*-test. ## $p < 0.01$ OA versus H-89 using a paired Student's *t*-test.

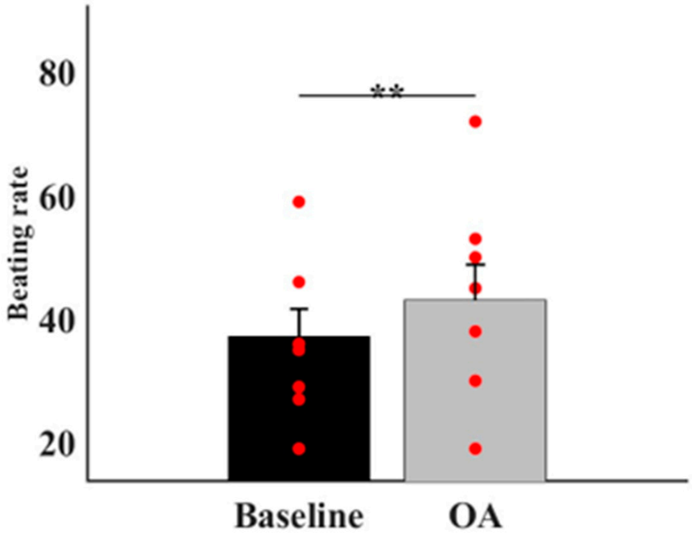

Figure S4. The effect of the PP2A inhibitor okadaic acid (OA) on the beating rate of spontaneously beating hiPSC-CMs. The mean beating rate of spontaneously beating hiPSC-CMs under baseline conditions and following treatment with the PP2A inhibitor okadaic acid (OA) (100 µM) (n = 4). The data are presented as the mean ± SEM. The statistical analysis was performed using a one-sample t-test versus baseline values, followed by one-way ANOVA with Dunn–Šidák multiple comparison post hoc analysis. ** $p < 0.01$ versus the baseline.

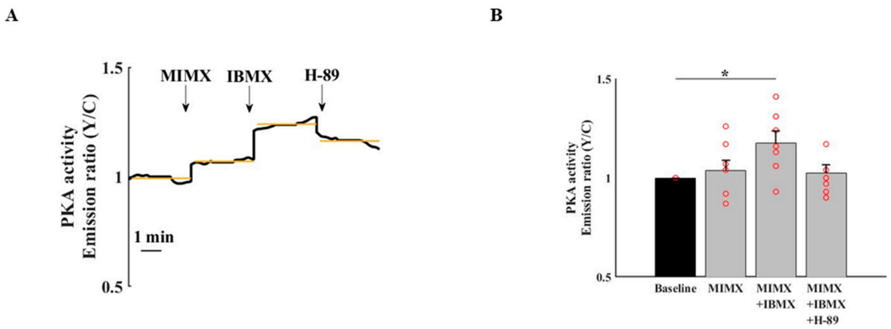

Figure S5. The effect of the PDE1 inhibitor MIMX on PKA activity in the cytosol compartment of spontaneously beating hiPSC-CMs. (**A**) A representative example of cytosol compartment PKA activity in a single spontaneously beating hiPSC-CM in response to the PDE1 inhibitor MIMX (50 µM), followed by the PDE inhibitor (IBMX) at 100 µM and PKA inhibition with 10 µM H-89. (**B**) The mean PKA activity in the cytosol compartment in a single spontaneously beating hiPSC-CM in response to the PDE1 inhibitor MIMX (50 µM), followed by the PDE inhibitor (IBMX) at 100 µM and PKA inhibition with 10 µM H-89 (n = 7). The data are presented as the mean ± SEM. * $p < 0.05$ MIMX, IBMX, or H-89 compared with a value of 1.0 using a one-sample t-test. Compared among MIMX, IBMX, or H-89 using a one-way ANOVA with Dunn–Šidák multiple comparison.

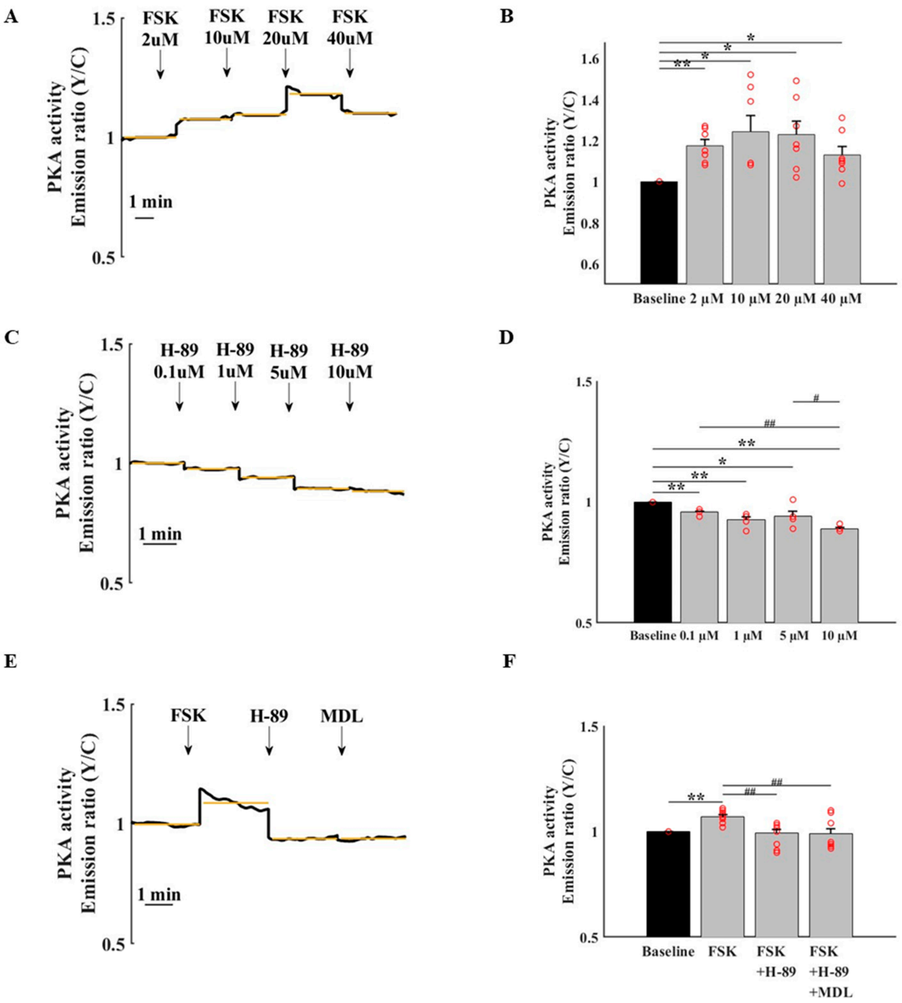

Figure S6. The effect of the adenylyl cyclase (AC) activator (forskolin) or inhibitor (H-89) on PKA activity in the mitochondrial matrix (MM) of spontaneously beating hiPSC-CMs. (**A**) A representative example of MM PKA activity (4Cox8-AKAR4 emission Y/C) in a single hiPSC-CM in response to increasing concentrations of forskolin (FSK) (2, 10, 20 and 40 μM). (**B**) The mean PKA activity in the MM (4Cox8-AKAR4 emission Y/C) in a single hiPSC-CM to increasing concentrations of forskolin (FSK) (2, 10, 20, and 40 μM ($n = 7$). (**C**) A representative example of MM PKA activity (4Cox8-AKAR4 emission Y/C) in a single hiPSC-CM in response to treatment with increasing concentrations of the PKA inhibitor H-89 (0.1, 1, 5, and 10 μM). (**D**) The mean PKA activity in the MM in response to increasing concentrations of H-89 ($n = 5$). (**E**) A representative example of MM PKA activity (4Cox8-AKAR4 emission Y/C) in a single hiPSC-CM in response to treatment with FSK and H-89 by the addition of the adenyl cyclase inhibitor MDL (400 μM). (**F**) The mean PKA activity in the MM (4Cox8-AKAR4 emission Y/C) in a single hiPSC-CM in response to 20 μM FSK and 10 μM H-89, followed by the addition of MDL ($n = 10$). The data are presented as the mean ± SEM. * $p < 0.05$ and ** $p < 0.01$ FSK, H-89, and MDL responses compared with a value of 1.0 using a one-sample t-test. # $p < 0.05$ and ## $p < 0.01$compared among H-89 responses/FSK/FSK + H89 using a one-way ANOVA with Dunn–Šidák multiple comparison.

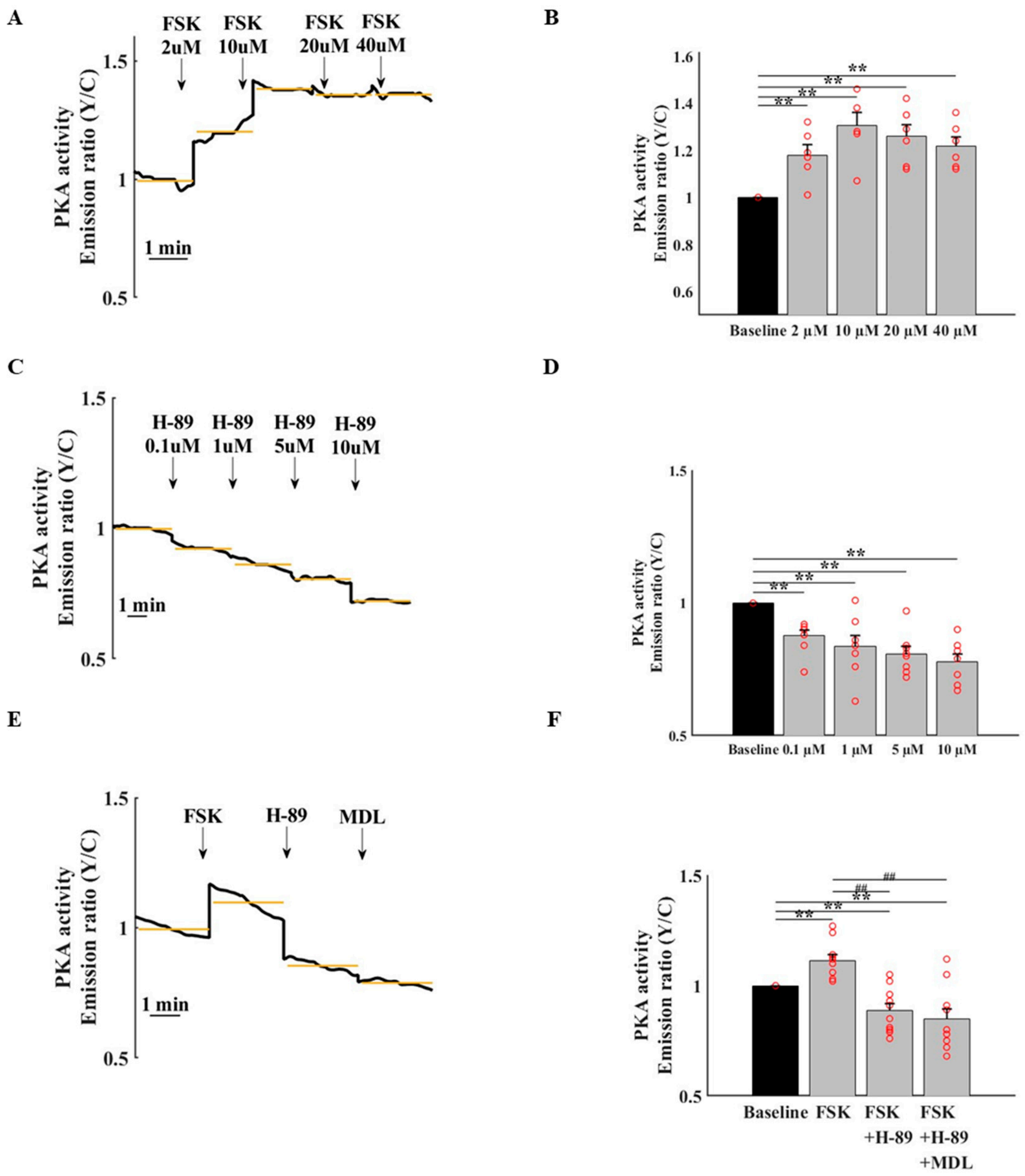

Figure S7. The effect of the adenylyl cyclase (AC) activator (forskolin) or inhibitor (H-89) on PKA activity in the OMM of spontaneously beating hiPSC-CMs. (**A**) A representative example of OMM PKA activity (yTom-AKAR4 emission Y/C) in a single hiPSC-CM in response to increasing concentrations of forskolin (FSK) (2, 10, 20, and 40 μM). (**B**) The mean PKA activity in the OMM (yTom-AKAR4 emission Y/C) in a single hiPSC-CM in response to increasing concentrations of forskolin (FSK) (2, 10, 20, and 40 μM) (n = 6). (**C**) A representative example of OMM PKA activity (yTom-AKAR4 emission Y/C) in a single hiPSC-CM in response to treatment with increasing concentrations of the PKA inhibitor H-89 (0.1, 1, 5, and 10μM). (**D**) The mean PKA activity in the OMM (yTom-AKAR4 emission Y/C) in a single hiPSC-CM in response to treatment with increasing concentrations of the PKA inhibitor H-89 (n = 8). (**E**) A representative example of MM PKA activity (yTom-AKAR4 emission Y/C) in a single hiPSC-CM in response to treatment with FSK and H-89 by addition of the adenyl cyclase inhibitor MDL (400 μM). (**F**) The mean PKA activity in the MM (yTom-AKAR4 emission Y/C) in a single hiPSC-CM in response to 20 μM FSK and 10 μM H-89, followed by

addition of MDL (n = 10). The data are presented as the mean ± SEM. ** $p < 0.01$, FSK, and H-89 responses compared with a value of 1.0 using a one-sample t-test. ## $p < 0.01$compared among H-89 responses using a one-way ANOVA with Dunn–Šidák multiple comparison.

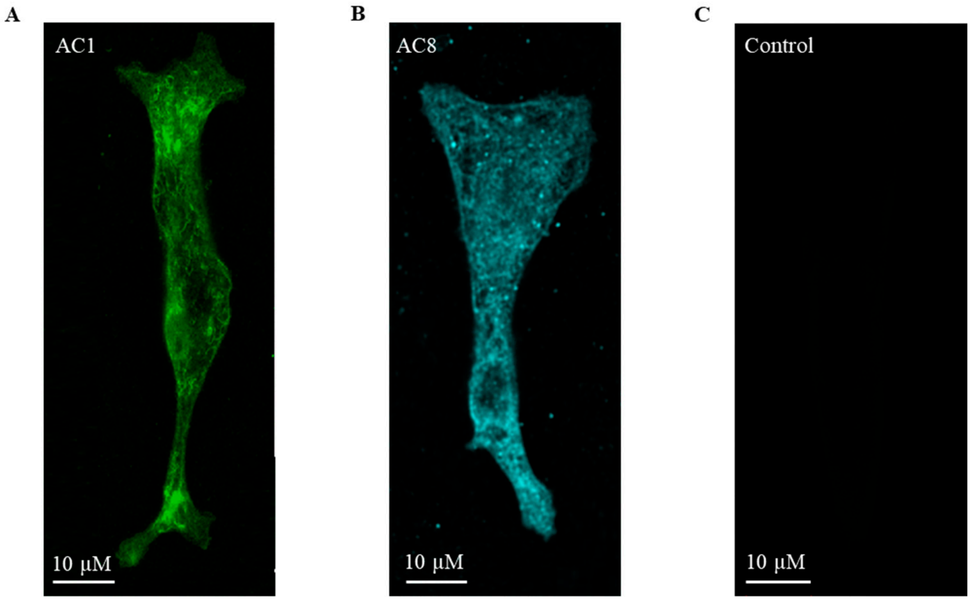

Figure S8. Immunofluorescence staining of Ca^{2+}-activated adenylyl cyclase (AC) in a single hiPSC-CM. Images of immunofluorescence staining of AC1 with an anti-AC1 antibody (**A**), AC8 with an anti-AC8 antibody in hiPSC-CMs, and (**B**) the control with a secondary antibody only (**C**).

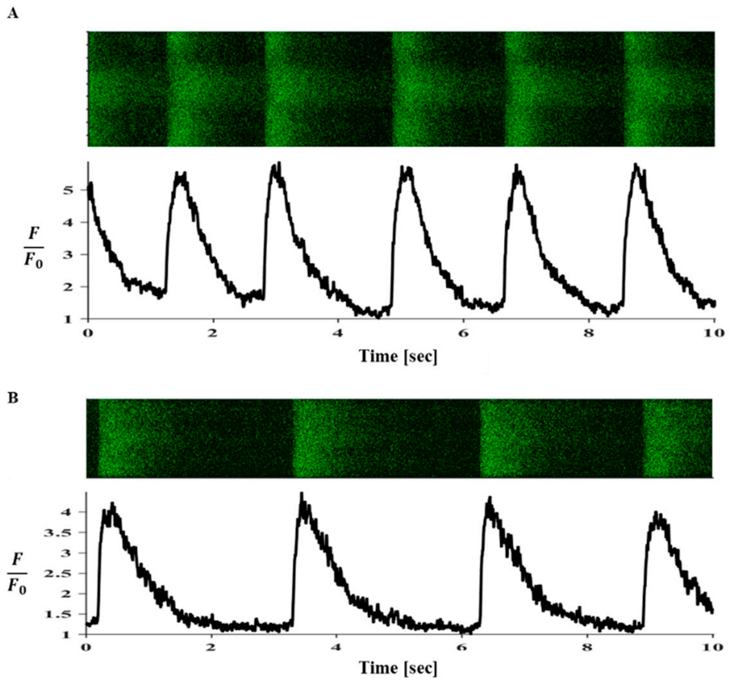

Figure S9. The effects of BAPTA on the beating rate of hiPSC-CMs. (**A**, **B**) Representative confocal line-scan images prior to (**A**) and following exposure to 25 µM BAPTA.

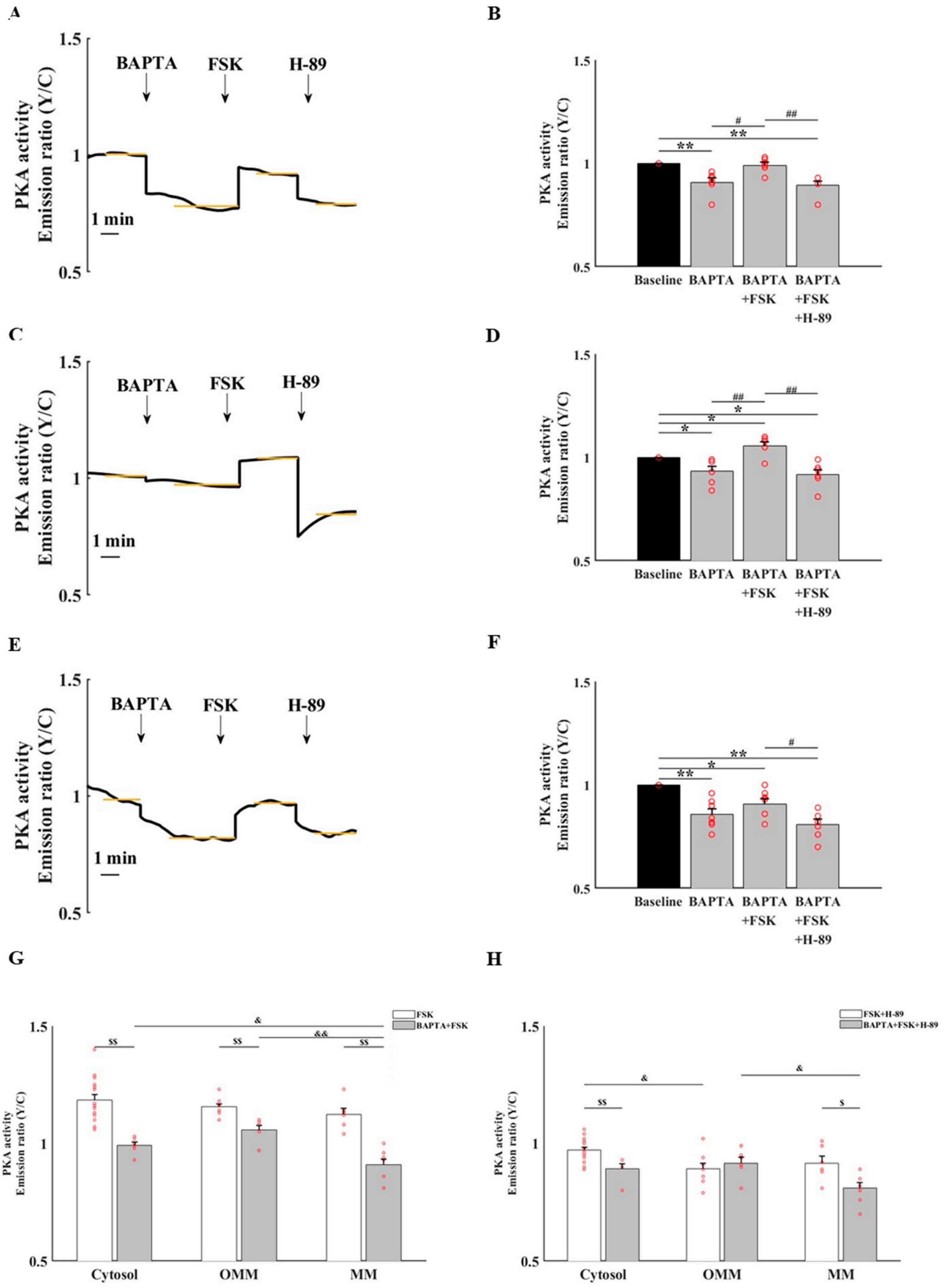

Figure S10. PKA activity in the cytosol and in mitochondrial compartments of spontaneously beating hiPSC-CMs. (**A**) A representative example of cytosolic PKA activity (AKAR4 emission Y/C) in a single hiPSC-CM in response to increasing concentrations of a Ca^{2+} chelator, BAPTA (25 μM), after 5 min recording, followed by adenylyl cyclase (AC) activation using 20 μM forskolin (FSK) and PKA inhibition using 10 μM H-89 after 3 min recording for each. (**B**)

The mean PKA activity in the cytosol in response to increasing concentrations of BAPTA, FSK, and H-89 ($n = 6$). (**C**) AS representative example of PKA activity in the outer mitochondrial membrane (OMM, yTom-AKAR4 emission Y/C) in a single hiPSC-CM in response to increasing concentrations of BAPTA (25 μM), followed with 20 μM FSK and 10 μM H-89. (**D**) The mean PKA activity in the OMM in response to increasing concentrations of BAPTA, FSK, and H-89 ($n = 6$). (**E**) A representative example of mitochondrial matrix (MM) PKA activity (4Cox8-AKAR4 emission Y/C) in a single hiPSC-CM in response to increasing concentrations of BAPTA (25 μM), followed with 20 μM FSK and 10 μM H-89. (**F**) The mean PKA activity in the MM in response to increasing concentrations of BAPTA, FSK, and H-89 ($n = 7$). (**G**) PKA activity in the cytosol, OMM, and MM of single hiPSC-CM following coadministration of BAPTA (25 μM) and FSK (20 μM). (**H**) PKA activity in the cytosol, OMM, and MM of single hiPSC-CM following coadministration of BAPTA (25 μM) and H-89 (10 μM). The data are presented as the mean± SEM. $^{*}p < 0.05$ and $^{**}p < 0.01$ BAPTA, FSK, or H-89 compared with a value of 1.0 using a one-sample t-test. # $p < 0.05$ ## $p < 0.01$ compared among BAPTA, FSK, and H-89 using a one-way ANOVA with Dunn–Šidák multiple comparison. $ < 0.05 and $$ < 0.01 FSK or H-89 versus the response in the presence of BAPTA in the same compartment using an unpaired Student's t-test. & p < 0.05, && p < 0.01 compared among H-89 responses in the cytosol, OMM, and MM using a one-way ANOVA with Dunn–Šidák multiple comparison.

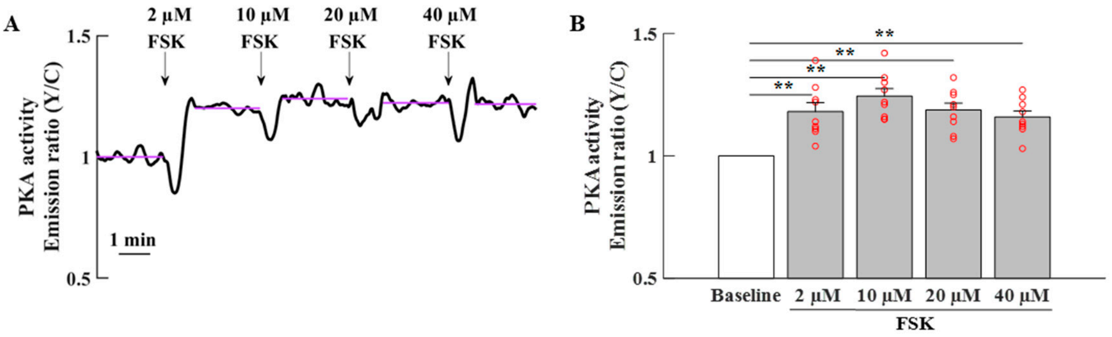

Figure S11. The effect of an AC activator (forskolin) on PKA activity in the cytosol of spontaneously beating SANCs. (**A**) A representative example of cytosolic PKA activity (AKAR4 emission Y/C) in a single pacemaker cell in response to increasing concentrations of forskolin (FSK) (2, 10, 20, and 40 μM) ($n = 9$). (**B**) The mean PKA activity in the cytosol in response to increasing concentrations of FSK. The data are presented as the mean ± SEM. ** $p < 0.01$ FSK responses compared with a value of 1.0 using a one-sample t-test. # $p < 0.05$ compared among FSK responses using a one-way ANOVA with Dunn–Šidák multiple comparison.

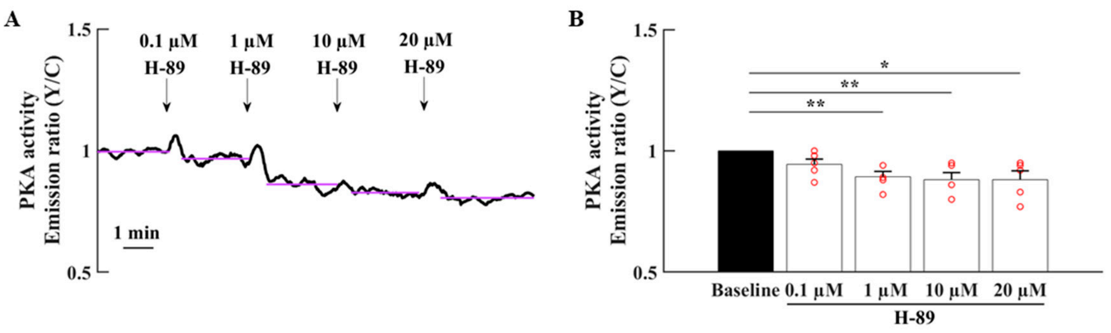

Figure S12. The effect of PKA inhibition (H-89) on PKA activity in the cytosol of spontaneously beating SANCs. (**A**) A representative example of cytosolic PKA activity (AKAR4 emission Y/C) in a single pacemaker cell in response to treatment with increasing concentrations of the PKA inhibitor H-89 (0.1, 1, 10, and 20 μM). (**B**) The mean PKA activity in the cytosol in response to increasing concentrations of H-89 ($n = 5$). The data are presented as the mean ± SEM. * $p < 0.05$. ** $p < 0.01$ H-89 responses compared with a value of 1.0 using a one-sample t-test.

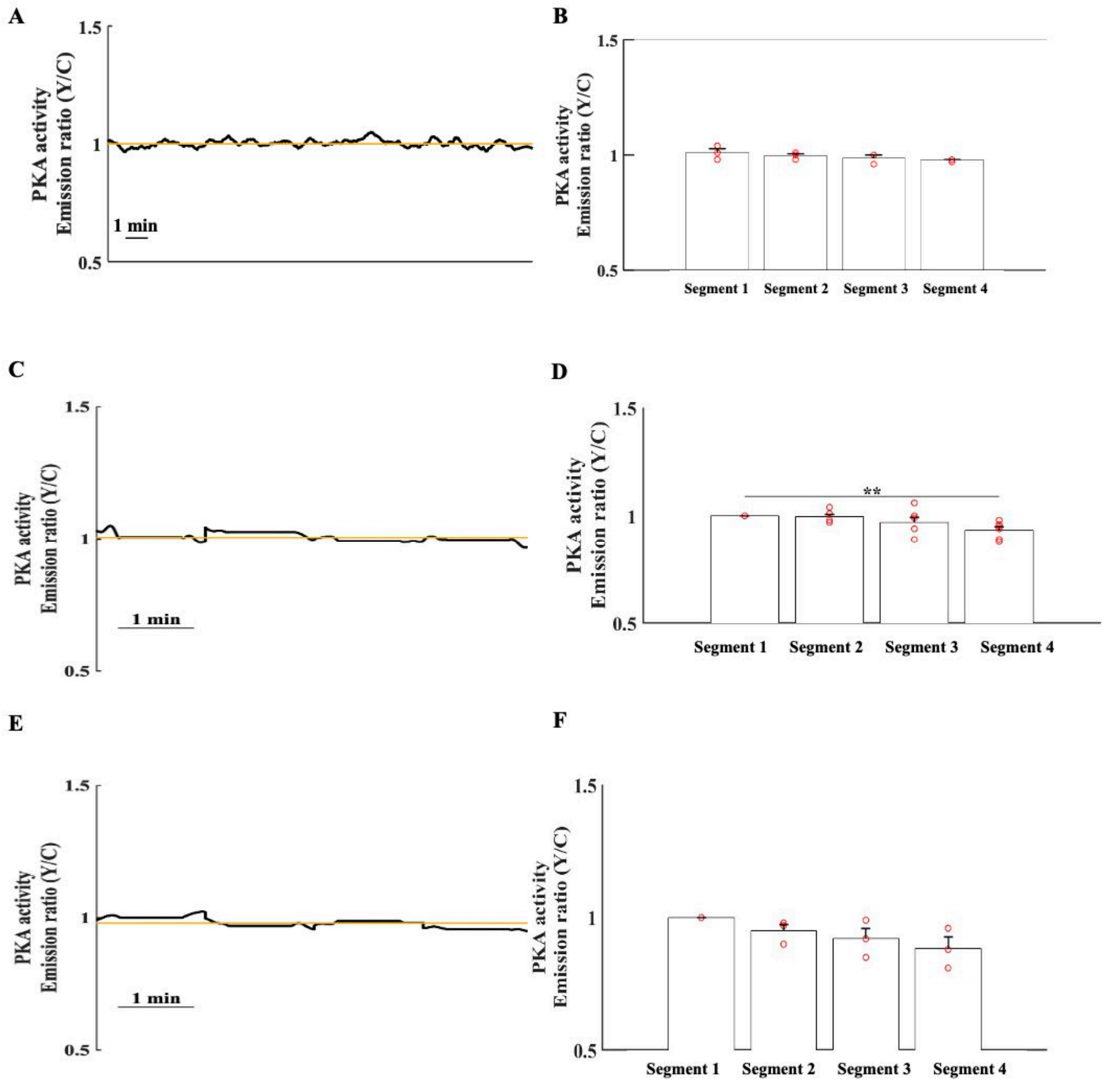

Figure S13. Time-control measurements of PKA activity in spontaneously beating hiPSC-CMs. (**A**) A representative example of cytosolic PKA activity (AKAR4

emission Y/C) in a single hiPSC-CM measured for 20 min in the presence of DMSO. (**B**) The mean PKA activity measurements in the cytosol (AKAR4 emission Y/C) were divided into 5 min segments for comparison and assessment of decay (n = 3). No significant decay in fluorescence was observed. (**C**) A representative example of mitochondrial matrix (MM) PKA activity (4Cox8-AKAR4 emission Y/C) in a single hiPSC-CM measured for 20 min. (**D**) The mean PKA activity measurements in the MM (4Cox8-AKAR4 emission Y/C) were divided into 5 min segments for comparison and assessment of decay (n = 6). No significant decay in fluorescence was observed. (**E**) A representative example of outer mitochondrial membrane (OMM) PKA activity (yTom-AKAR4 emission Y/C) in a single hiPSC-CM measured for 20 min. (**F**) The mean PKA activity measurements in the OMM (yTom-AKAR4 emission Y/C) were divided into 5 min segments for comparison and assessment of decay (n = 3). No significant decay in fluorescence was observed. **$p < 0.01$ using one-way ANOVA between adjacent segments (one after the other).

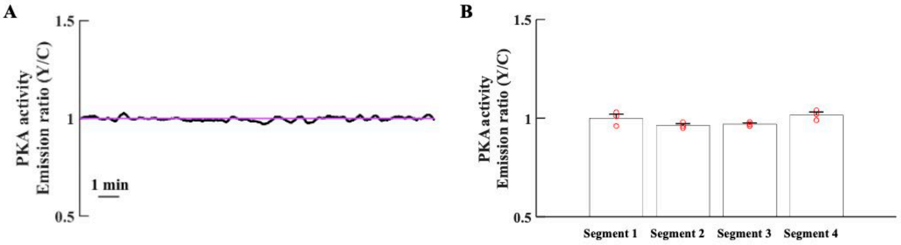

Figure S14. Time-control measurements of PKA activity in spontaneously beating SANCs in the presence of DMSO. (**A**) A representative example of cytosolic PKA activity (AKAR4 emission Y/C) in a single pacemaker cell measured for 20 min. (**B**) The mean PKA activity measurements in the cytosol were divided into 5 min segments for comparison and assessment of decay (n = 3). No significant decay in fluorescence was observed.

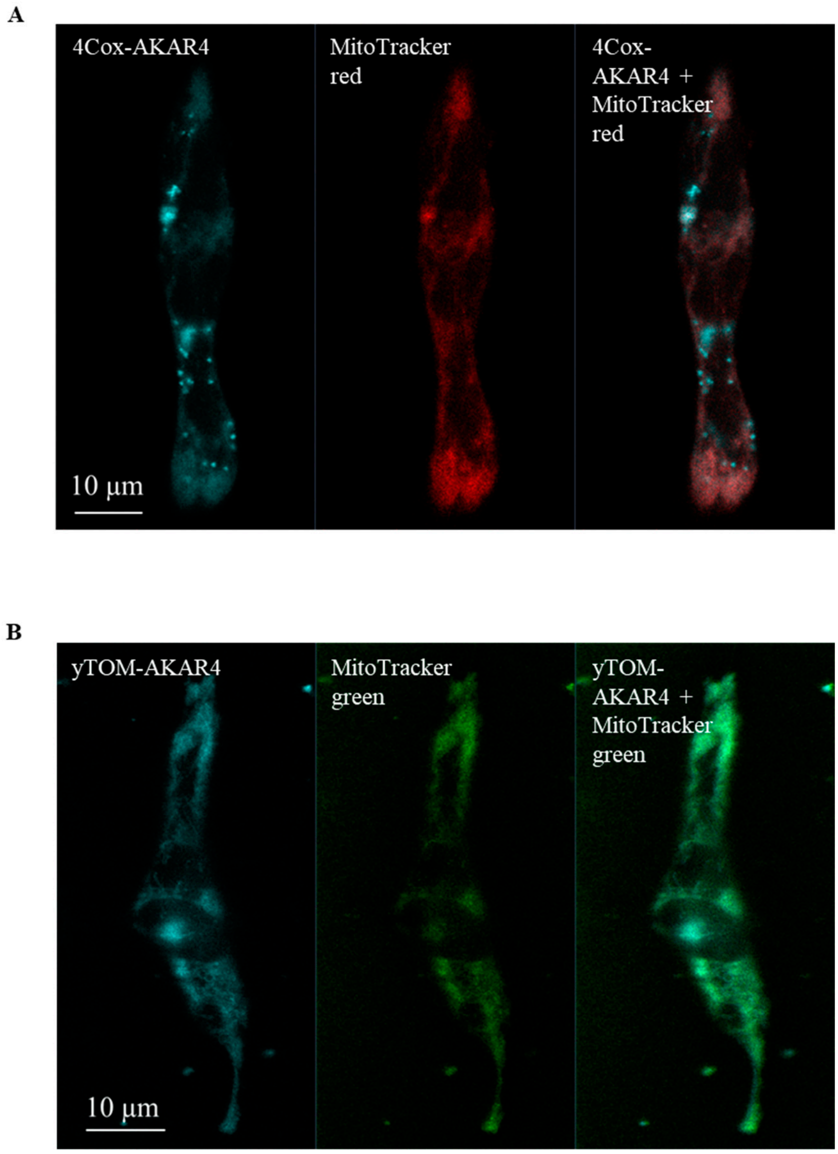

Figure S15. Localization of 4Cox8-AKAR4 in the mitochondrial matrix (MM) and of yTom-AKAR4 in the outer mitochondrial membrane (OMM) of spontaneously beating hiPSC-CMs. (**A**) A confocal image of a single hiPSC-CM expressing 4Cox8-AKAR4 and loaded with MitoTracker red as the MM indicator, excited alone and together. (**B**) A confocal image of a single hiPSC-CM expressing yTom-AKAR4 and loaded with MitoTracker green as the OMM indicator, excited alone and together.

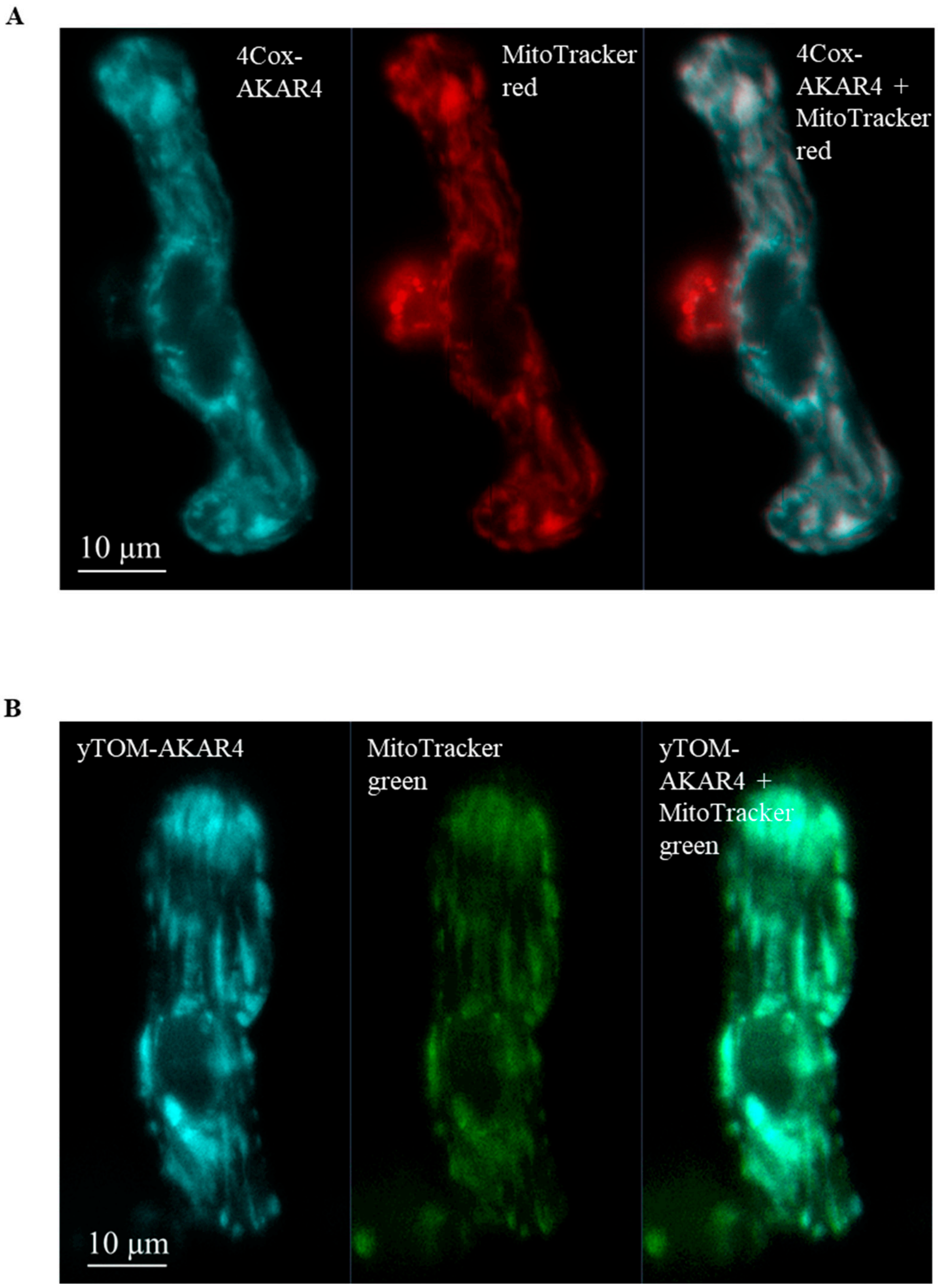

Figure S16. Localization of 4Cox8-AKAR4 in the membrane matrix (MM) and yTom-AKAR4 in the outer mitochondrial membrane (OMM) of spontaneously beating SANCs. (**A**) A confocal image of a single pacemaker cell expressing 4Cox8-AKAR4 and loaded with MitoTracker red as the MM indicator, excited alone and together. (**B**) A confocal image of a single pacemaker cell expressing yTom-AKAR4 and loaded with MitoTracker green as the OMM indicator, excited alone and together.

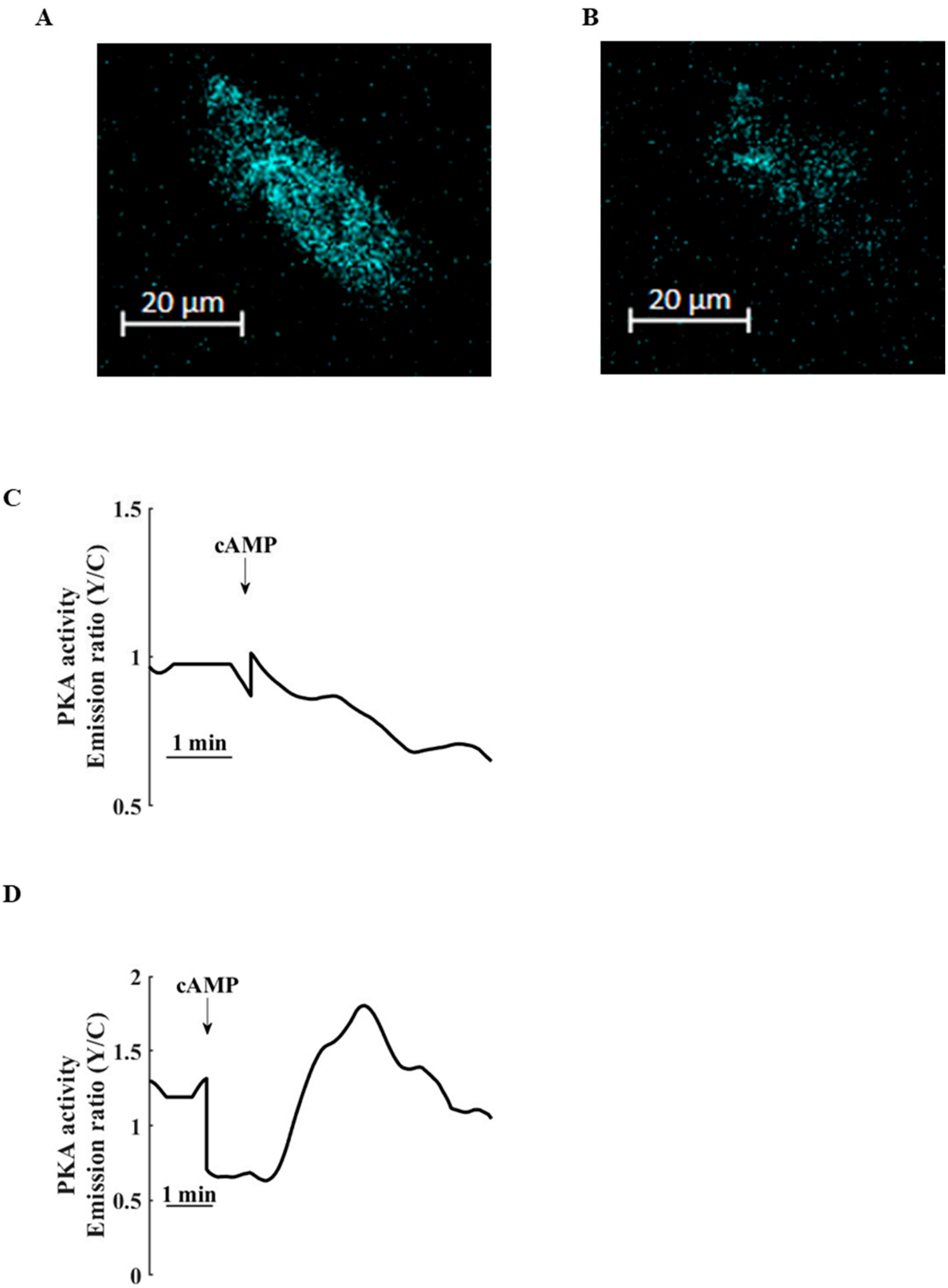

Figure S17. Localization of 4Cox8-AKAR4 in the mitochondrial matrix (MM) and of yTom-AKAR4 in the outer mitochondrial membrane (OMM) of spontaneously permeabilized beating hiPSC-CMs. A representative example of cytosolic PKA activity (AKAR4 emission Y/C) in a single hiPSC-CM, measured before (A) and after (B) the use of 5 μM digitonin. (C) A representative example of PKA activity (4Cox8-AKAR4 emission Y/C) in a single hiPSC-CM using 25 μM digitonin in the presence of 10 μM cAMP. (D) A representative example of PKA activity (yTom-AKAR4 emission Y/C) in a single hiPSC-CM using 25 μM digitonin in the presence of 10 μM cAMP.
